# Supplementary material for: A high-speed search engine pLink 2 with systematic evaluation for proteome-scale identification of cross-linked peptides
Source: Nat Commun. 2019 Jul 30;10:3404. doi: 10.1038/s41467-019-11337-z (PMC6667459; doi:10.1038/s41467-019-11337-z)
Supplement: Supplementary file 1 — Supplementary Information [file 41467_2019_11337_MOESM1_ESM.pdf]

Supplementary Information

A high-speed search engine pLink 2 with systematic evaluation for proteome-scale identification of cross-linked peptides

Chen et al.

Supplementary File	Title
Supplementary Table 1	The ten search engines used in this study
Supplementary Table 2	Detailed information of the twelve datasets used in this study
Supplementary Table 3	The performance of ten search engines on the Simulated-SS dataset
Supplementary Table 4	Search parameters on two synthetic datasets
Supplementary Table 5	Search parameters on two ^{15}N metabolically labeled datasets
Supplementary Table 6	Search parameters on two datasets used in entrapment database method
Supplementary Table 7	Nine features used in pLink 2 for training the SVM classifier
Supplementary Fig. 1	Performance comparison on the HeLa cell dataset of the Xolik paper
Supplementary Fig. 2	The sensitivity and precision of Kojak, pLink 1, and pLink 2 on the Synthetic-BS3 dataset
Supplementary Fig. 3	Performance evaluation on the Synthetic-SS dataset
Supplementary Fig. 4	The sensitivity and precision of Kojak, pLink 1, and pLink 2 on the Synthetic-SS dataset
Supplementary Fig. 5	Demonstration of result validation using ^{15}N metabolically labeled datasets
Supplementary Fig. 6	The performances of two ^{15}N-labeling experiments
Supplementary Fig. 7	Analysis of NaN-ratio results on the E.coli-Leiker-^{15}N dataset
Supplementary Fig. 8	Compare pLink 2 with Protein Prospector (PP) on the E.coli-Leiker-^{15}N dataset
Supplementary Fig. 9	Performance evaluation on the E.coli-SS-^{15}N dataset
Supplementary Fig. 10	Analysis of NaN-ratio results on the E.coli-SS-^{15}N dataset
Supplementary Fig. 11	Comparison of cross-linked residue pairs identified by pLink 1 between this study and the publication of Kojak
Supplementary Fig. 12	Performance evaluation on the Ca_V1.1-SS dataset
Supplementary Fig. 13	Distribution of precursor masses on the Synthetic-BS3 and the Synthetic-SS datasets
Supplementary Fig. 14	The speedup of pLink 2 on the Synthetic-SS dataset
Supplementary Fig. 15	The potential of using tag-based indexing to accelerate cross-linked peptide identification
Supplementary Fig. 16	The identification rate and the proportion of identified cross-linked scans in all identified scans on four previously published datasets
Supplementary Fig. 17	The scheme of constructing a fragment index
Supplementary Note 1	Evaluate the performance of ten search engines using synthetic datasets
Supplementary Note 2	The relationship between the global FDR and subgroup FDRs of intra-protein and inter-protein cross-linked identifications
Supplementary Note 3	Deducing an NaN-FDR for a search engine independent of the TDA-FDR
Supplementary Note 4	Evaluate the performance of XlinkX
Supplementary Note 5	Evaluate the performance of MassSpecStudio 2
Supplementary Note 6	Evaluate the acceleration effect of fragment indexing
Supplementary Note 7	Application of pLink 2 at a proteome scale
Supplementary Note 8	The method used to generate simulated spectra

Supplementary Table 1. The ten search engines used in this study

Search Engine	Website	Version	Search Strategy[a]	Publication Year	Reference
xQuest / xProphet	http://proteomics.ethz.ch/cgi-bin/xquest2_cgi/download.cgi	2.1.1	E / O	2008 / 2012	[1, 2]
StavroX	http://www.stavrox.com/Download.htm	3.6.0.1	E	2012	[3]
pLink 1 (pLink / pLink-SS)	http://pfind.ict.ac.cn/software/pLink1/index.html	1.23	E / O	2012 / 2015	[4, 5]
Protein Prospector	http://prospector.ucsf.edu/prospector/mshome.htm	v5.21.2	O	2014	[6]
Kojak	http://www.kojak-ms.org/download.html	1.5.5	O	2015	[7]
Xi	https://github.com/Rappsilber-Laboratory/XiSearch	1.6.731	O	2016 / 2019	[8, 9]
Xilmass	https://github.com/compomics/xilmass	1.0	E	2016	[10]
MetaMorpheusXL	https://github.com/smith-chem-wisc/MetaMorpheus/releases	0.0.285	O	2018	[11]
Xolik	http://bioinformatics.ust.hk/Xolik.html	0.3	E	2018	[12]
pLink 2	http://pfind.ict.ac.cn/software/pLink/index.html	2.2	O	-	[13]

a E for exhaustive search and O for open search.

Supplementary Table 2. Detailed information of the twelve datasets used in this study

Dataset	Cross-linker	Mass spectrometer	# Files (RAW / MGF)	# MS2 scans	Reference
Simulated-BS3	BS3	-	1	10,000	-
Simulated-SS	SS	-	1	10,000	-
Synthetic-BS3	BS3	LTQ-Orbitrap-ETD	1	2,077	[4]
Synthetic-SS	SS	LTQ-Orbitrap-ETD	1	5,000	[5]
E.coli-Leiker-^{15}N	Leiker	Q Exactive HF	5	258,555	-
E.coli-SS-^{15}N	SS	Q Exactive HF	10	289,381	-
SCF(FBXL3)-BS3	BS3	Q Exactive	3	81,300	[7]
Ca_v1.1-SS	SS	Q Exactive	2	95,339	[14]
E.coli-Leiker	Leiker	Q Exactive	116	2,144,734	[15]
C.elegans-Leiker	Leiker	Q Exactive	36	687,916	[15]
E.coli-SS	SS	Q Exactive	98	2,848,512	[5]
Human-SS	SS	Q Exactive	32	966,351	[5]

Supplementary Table 3. The performance of ten search engines on the Simulated-SS dataset[a]

Search engine	Search strategy	Sensitivity (%)	Precision (%)	Run time (Min)	Selected
xQuest[b]	Exhaustive	-	-	-	No
Xilmass[c]	Exhaustive	-	-	-	No
Xolik	Exhaustive	19.9	97.3	0.4	No
MetaMorpheusXL	Open	57.8	98.1	0.2	No
StavroX	Exhaustive	61.1	47.5	42.6	No
Protein Prospector	Open	64.5	97.9	18.8	No
Xi	Open	68.6	95.5	0.9	No
Kojak	Open	79.7	96.7	1.2	Yes
pLink 1	Open	99.8	92.6	8.9	Yes
pLink 2	Open	99.9	100.0	0.7	Yes

a For sensitivity, precision, and run time, the average values obtained using three randomly generated Simulated-SS datasets were shown.

b xQuest did not report any results.

c Xilmass did not support the disulfide bond cross-linker and could not set user defined cross-linkers.

Supplementary Table 4. Search parameters on two synthetic datasets

Parameters	Synthetic-BS3	Synthetic-SS
Cross-linker	BS3	SS
Enzyme	Trypsin	Trypsin
Max Missed Cleavage Sites	2	4
Peptide Mass Range	[500, 6,000]	[500, 6,000]
Peptide Length Range	[5, 60]	[5, 60]
Precursor Tolerance	±20ppm	±20ppm
Fragment Tolerance	±20ppm	±20ppm
Fixed Modifications	Carbamidomethyl[C]	-
Variable Modifications	-	Trioxidation[C]
Max Modified Sites	-	3
Filter Tolerance	±10ppm	±10ppm
FDR	5% at PSM level	5% at PSM level

Supplementary Table 5. Search parameters on two ^{15}N metabolically labeled datasets

Parameters	E.coli-Leiker-^{15}N	E.coli-SS-^{15}N
Cross-linker	Leiker	SS
Enzyme	Trypsin	Glu-C and Trypsin
Max Missed Cleavage Sites	2	4
Peptide Mass Range	[600, 6,000]	[500, 6,000]
Peptide Length Range	[6, 60]	[5, 60]
Precursor Tolerance	±20ppm	±20ppm
Fragment Tolerance	±20ppm	±20ppm
Fixed Modifications	Carbamidomethyl[C]	-
Variable Modifications	Oxidation[M]	Nethylmaleimide[C]
Max Modified Sites	3	3
Filter Tolerance	±10ppm	±10ppm
FDR	5% at PSM level	5% at PSM level

Supplementary Table 6. Search parameters on two datasets used in entrapment database method

Parameters	SCF(FBXL3)-BS3	Ca_v1.1-SS
Cross-linker	BS3	SS
Enzyme	Trypsin	Trypsin
Max Missed Cleavage Sites	4	3
Peptide Mass Range	[500, 8,000]	[600, 6,000]
Peptide Length Range	[5, 80]	[6, 60]
Precursor Tolerance	±20ppm	±20ppm
Fragment Tolerance	±20ppm	±20ppm
Fixed Modifications	Carbamidomethyl[C]	-
Variable Modifications	Oxidation[M]<br>Methyl[K]<br>Dimethly[K]<br>Trimethyl[K]<br>Acetyl[ProteinN-term]	Nethylmaleimide[C]<br>Deamidated[N]
Max Modified Sites	2	3
Filter Tolerance	±15ppm	±10ppm
FDR	5% at PSM level	5% at PSM level

Supplementary Table 7. Nine features used in pLink 2 for training the SVM classifier

	Feature	Meaning
Static	KSDPScore	Fine-score value
	AlphaIntRatio	The fraction of intensity of the peaks matched to α-peptide
	BetaIntRatio	The fraction of intensity of the peaks matched to β-peptide
	AlphaTagRatio	The maximum tag length in α-peptide / length of α-peptide
	BetaTagRatio	The maximum tag length in β-peptide / length of β-peptide
	ShortLen	Log(min(length of α-peptide, length of β-peptide))
	ScoreDiff	Difference in fine-score between the top match and the second match
Dynamic	ModRatio	Frequency of each modification or combinations in positive samples
	PrecursorErrFreq	Cumulative frequencies of precursor mass errors in 200 bins from –20 ppm to +20 ppm in positive samples

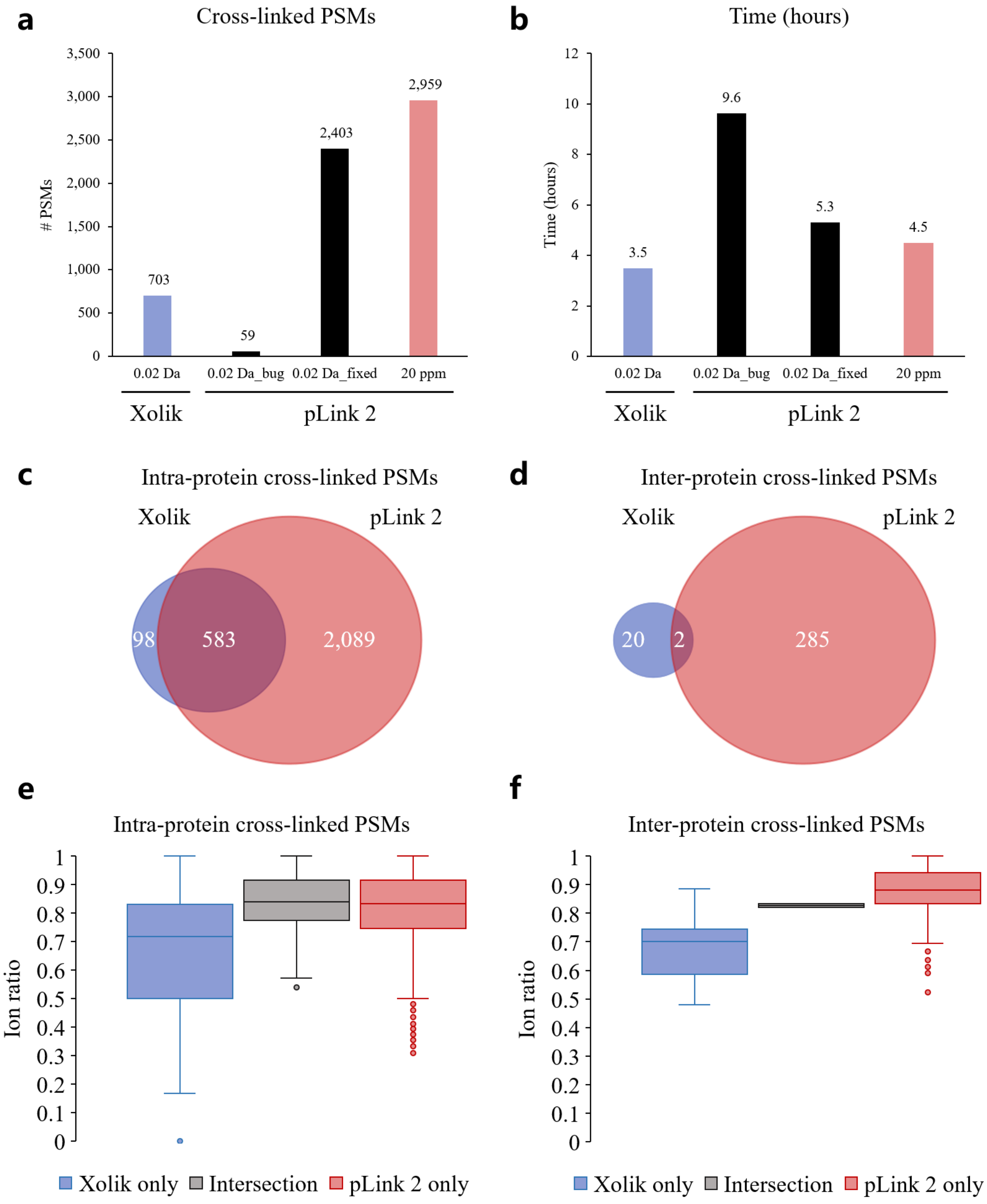

a
Cross-linked PSMs
PSMs
3,500
3,000
2,500
2,000
1,500
1,000
500
0
703
59
2,403
2,959
0.02 Da
0.02 Da_bug
0.02 Da_fixed
20 ppm
Xolik
pLink 2
b
Time (hours)
Time (hours)
12
10
8
6
4
2
0
3.5
9.6
5.3
4.5
0.02 Da
0.02 Da_bug
0.02 Da_fixed
20 ppm
Xolik
pLink 2
c
Intra-protein cross-linked PSMs
Xolik
pLink 2
98
583
2,089
d
Inter-protein cross-linked PSMs
Xolik
pLink 2
20
2
285
e
Intra-protein cross-linked PSMs
Ion ratio
1
0.9
0.8
0.7
0.6
0.5
0.4
0.3
0.2
0.1
0
Xolik only
Intersection
pLink 2 only
f
Inter-protein cross-linked PSMs
Ion ratio
1
0.9
0.8
0.7
0.6
0.5
0.4
0.3
0.2
0.1
0
Xolik only
Intersection
pLink 2 only

Supplementary Figure 1. Performance comparison on the HeLa cell dataset of the Xolik paper[12]. **a)** The identification results of cross-linked PSMs with FDR set at 5% at the PSM level. Xolik can only set Da as the unit of fragment tolerance, and pLink 2 had a bug when the unit of fragment tolerance was set as Da, which was why Xolik outperformed pLink 2 in terms of the number of identified PSMs in its paper. After the bug was fixed, pLink 2 identified many more cross-linked PSMs than Xolik. If the unit of fragment tolerance was set as ppm, which was recommended and had no bugs, pLink 2 identified four times as many cross-linked PSMs as Xolik. When using ppm as the unit of fragment tolerance, pLink 2 **b)** had a similar running time as Xolik and identified many more **c)** intra-protein PSMs and **d)** inter-protein PSMs than Xolik. For intra-protein PSMs, the identifications of pLink 2 covered more than 85% of that of Xolik. For inter-protein PSMs, there was small overlap between the identifications of pLink 2 and that of Xolik. Ion ratios of consistently and uniquely identified PSMs were calculated for **e)** intra-protein cross-links and **f)** inter-protein cross-links. The ion ratio, which equals to the number of matched ions divided by the total number of theoretical ions, is a search engine independent criterion to evaluate the quality of PSM. The ion ratios of PSMs unique to pLink 2 were closer to that of PSMs consistent to both search engines than that of PSMs unique to Xolik, indicating that the qualities of PSMs unique to pLink 2 were better than that of PSMs unique to Xolik. Box-plot elements in e) and f): center line, median; box limits, first and third quartile (Q1 and Q3); whiskers, from Q1–1.5 × IQR to Q3+1.5 × IQR; points, outliers.

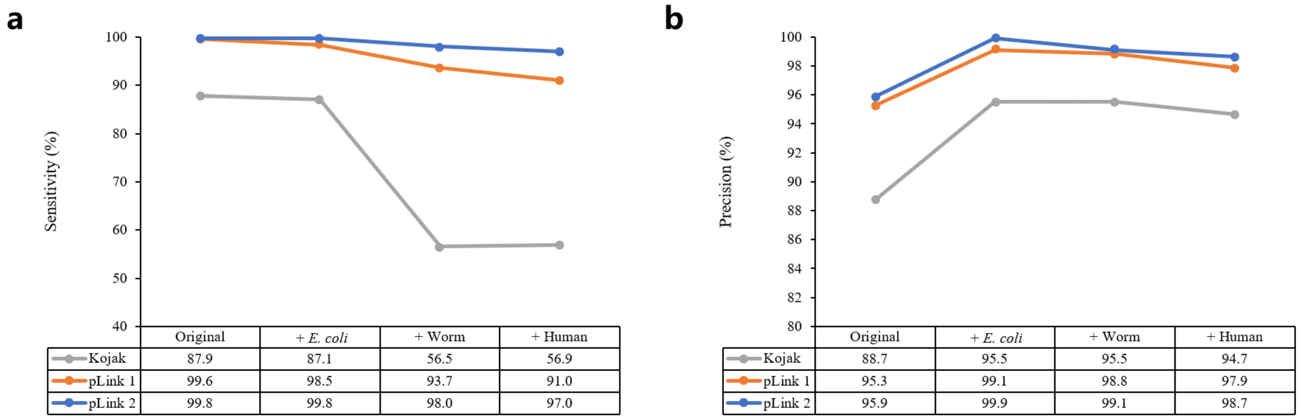

Supplementary Figure 2. Performance evaluation on the Synthetic-BS3 dataset. The **a)** sensitivity and **b)** precision of Kojak, pLink 1, and pLink 2 when searching against original database plus entrapment databases of different sizes.

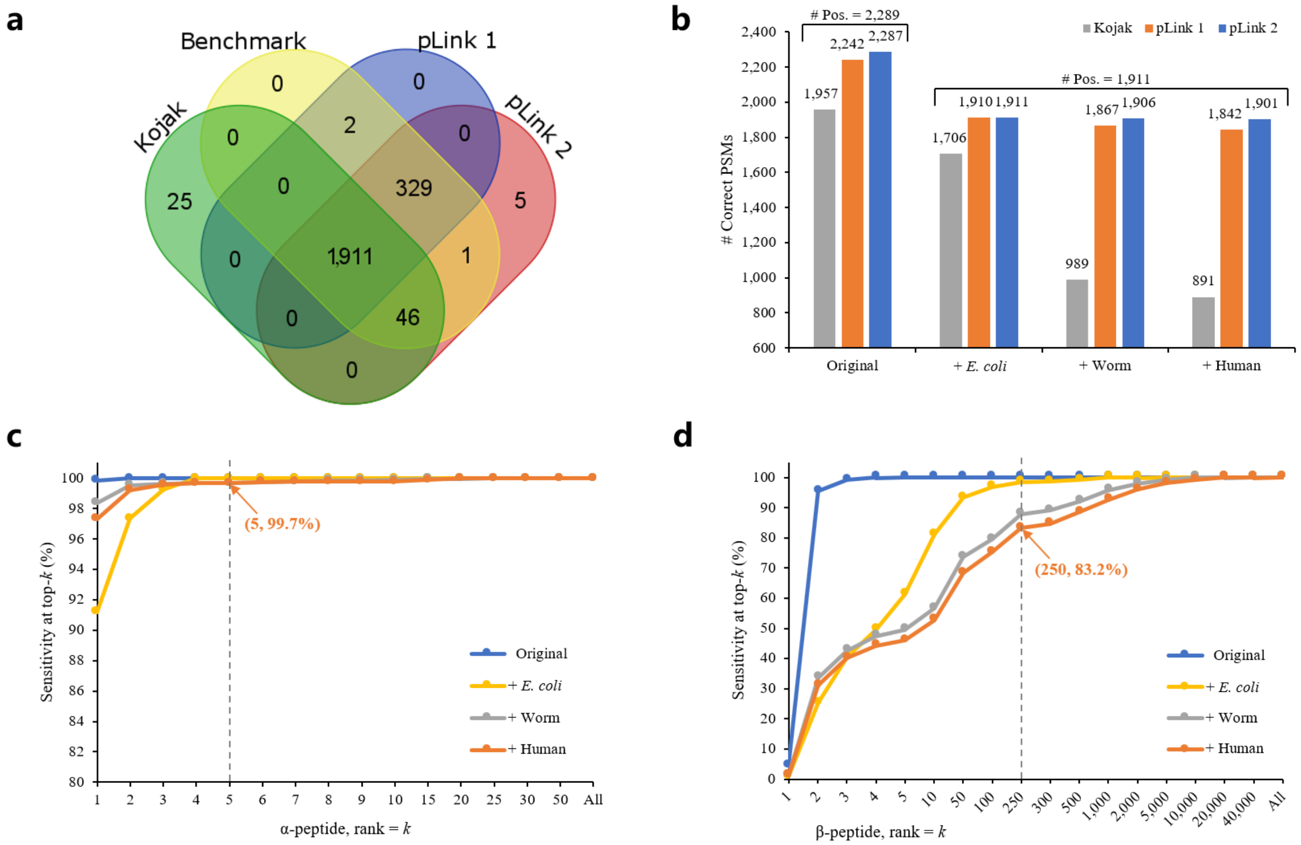

Supplementary Figure 3. Performance evaluation on the Synthetic-SS dataset. **a)** Venn diagram for the results of Kojak, pLink 1, pLink 2, and the benchmark. A total of 1,911 PSMs were correctly identified consistently by the three engines; these were used to be a new and fair standard dataset. **b)** The numbers of correctly identified PSMs by each search engine. **c)** The percentage of correct α-peptides ranking in the top-*k* in the open search stage of pLink 2. **d)** Similar to c), but for β-peptides. The "Original" database contains only the sequences of 72 synthetic peptides, "+ *E. coli*" database contains sequences from the "Original" database and the *E. coli* whole proteome database, and "+ Worm" and "+ Human" are similar to "+ *E. coli*".

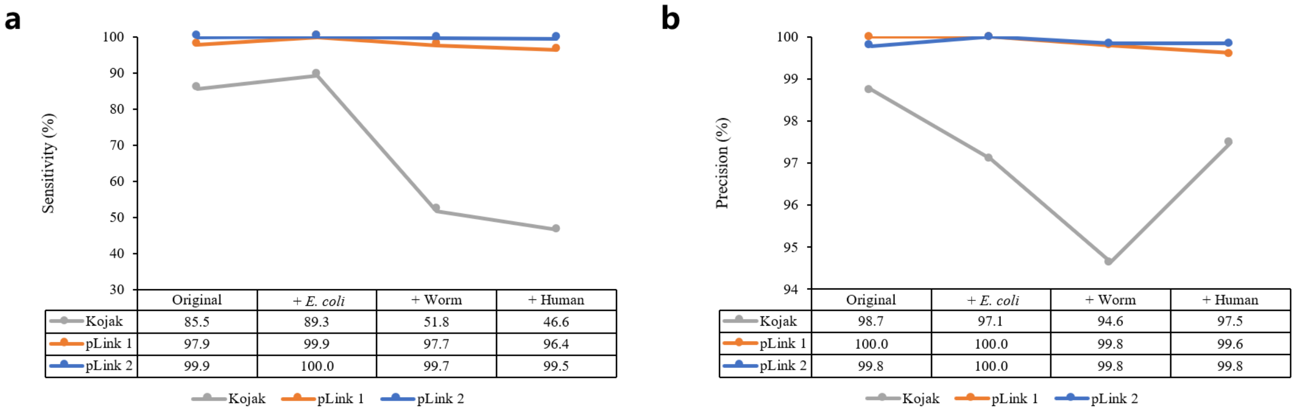

Supplementary Figure 4. Performance evaluation on the Synthetic-SS dataset. The **a)** sensitivity and **b)** precision of Kojak, pLink 1, and pLink 2 when searching against original database plus entrapment databases of different sizes.

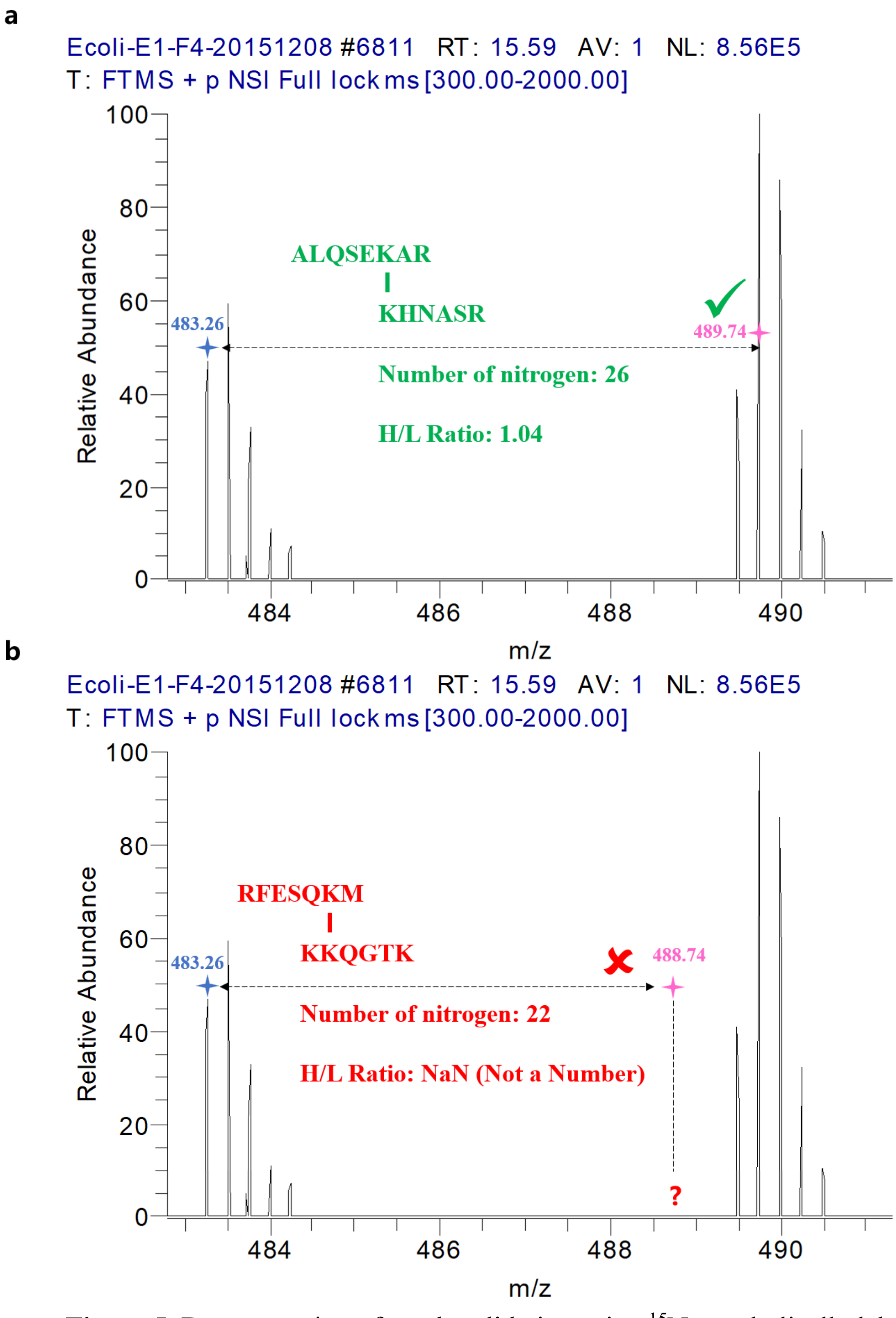

Supplementary Figure 5. Demonstration of result validation using ^{15}N metabolically labeled datasets. For the same MS2 scan of the E.coli-Leiker-^{15}N dataset, the cross-linked peptide pair identified by pLink 2 and Kojak was AIQSEKAR(6)-KHNASR(1) and RFESQKM(6)-KKQGTK(2) respectively. The MS1 scan was investigated by pQuant: **a)** the result of pLink 2 contains 26 nitrogen atoms, and the corresponding ^{15}N-labeled precursor ion was observed, resulting in a valid ^{15}N quantitation ratio of 1.04; **b)** the result of Kojak contains 22 nitrogen atoms, and the corresponding ^{15}N-labeled precursor ion was not observed, resulting in an invalid ^{15}N quantitation ratio as NaN (Not a Number).

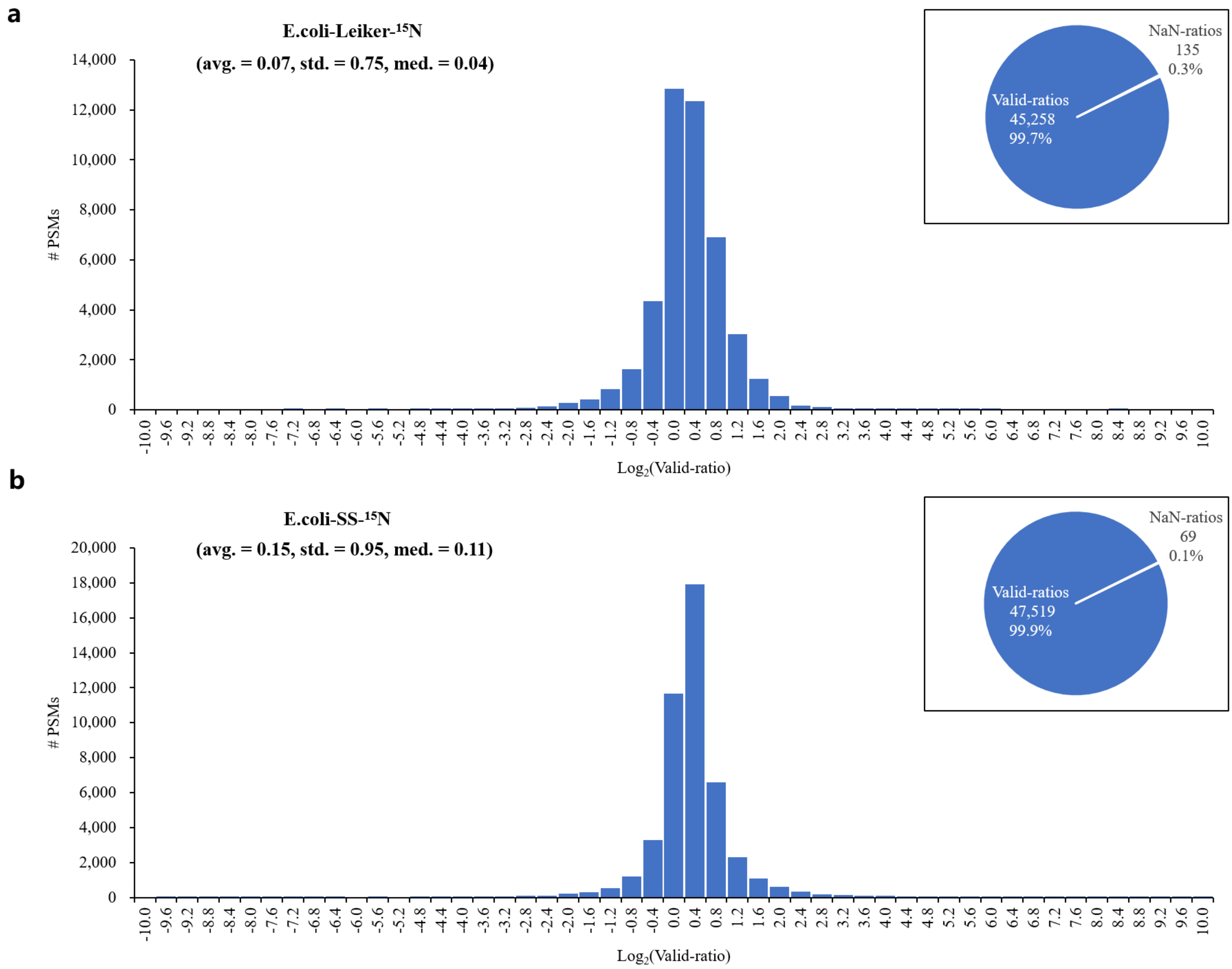

Supplementary Figure 6. The performances of the ^{15}N-labeling experiments. **a)** For the E.coli-Leiker-^{15}N dataset. **b)** For the E.coli-SS-^{15}N dataset. The performances of the ^{15}N-labeling experiments were evaluated by identifying and quantifying unlabeled regular peptides using pFind 3 and pQuant. pFind 3 was on restricted search mode and was configured with parameters in Supplementary Table 5 except that the FDR was set as 1% at peptide level. The main plots were histograms of log-transformed valid quantification ratios and the subplots were pie charts of valid-ratios and NaN-ratios.

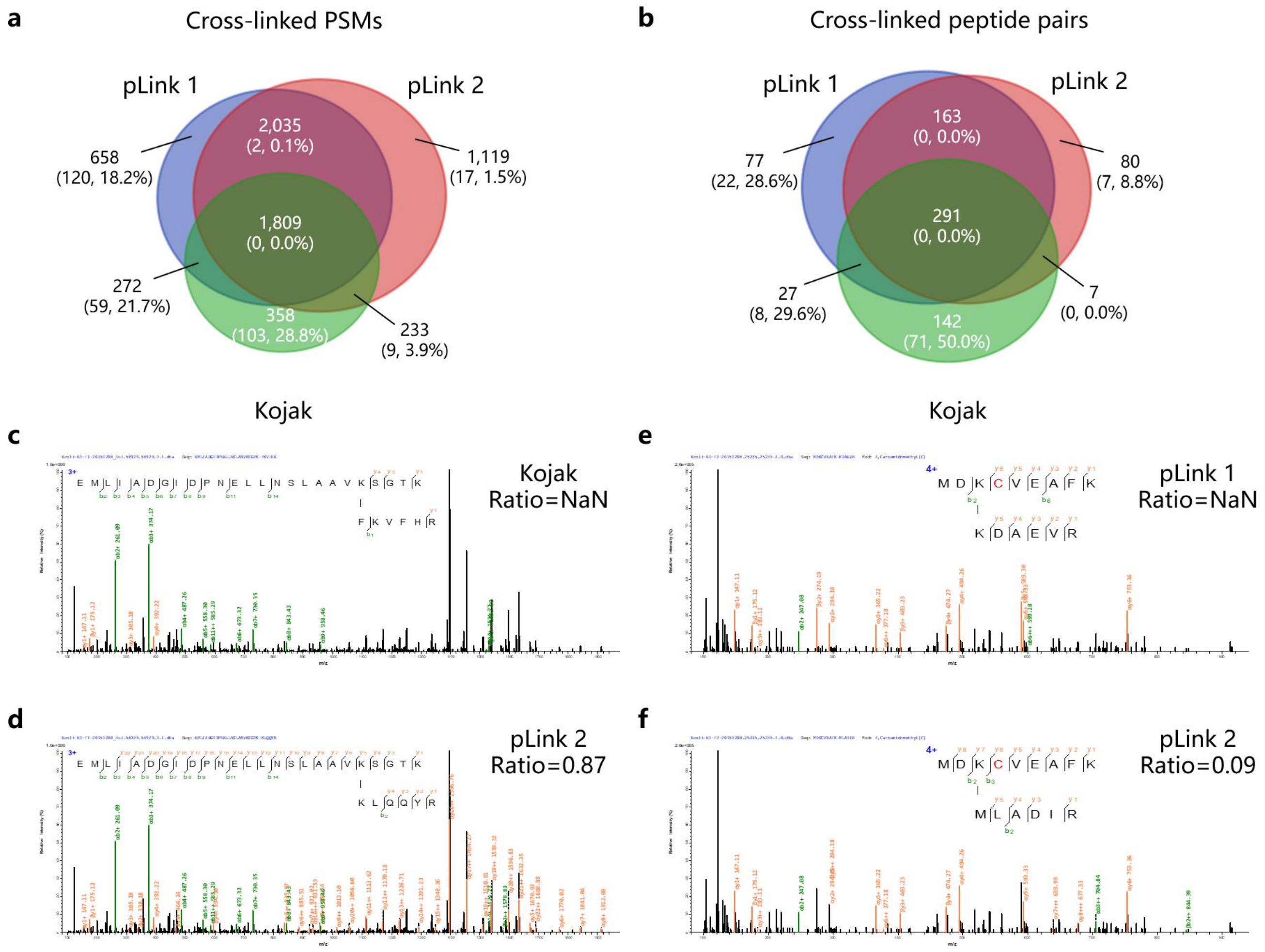

Supplementary Figure 7. Analysis of NaN-ratio results on the E.coli-Leiker-^{15}N dataset. **a)** PSMs and **b)** peptides uniquely identified by pLink 2 had the lowest percentage of NaN-ratios. Numbers in parentheses indicate the number and the percentage of NaN-ratio results that lie in the corresponding region. For example, 658 (120, 18.2%) means that pLink 1 uniquely identified 658 cross-linked PSMs, of which 120 were NaN-ratios, accounting for 18.2% of 658. An example of **c)** NaN-ratio peptide pair identified by Kojak and **d)** *non*-NaN-ratio peptide pair identified by pLink 2 for the same spectrum. An example of **e)** NaN-ratio peptide pair identified by pLink 1 and **f)** *non*-NaN-ratio peptide pair identified by pLink 2 for the same spectrum. A probable reason for the high percentage of NaN-ratios of Kojak and pLink 1 might be that they could not recall correct β-peptides in top-*k*.

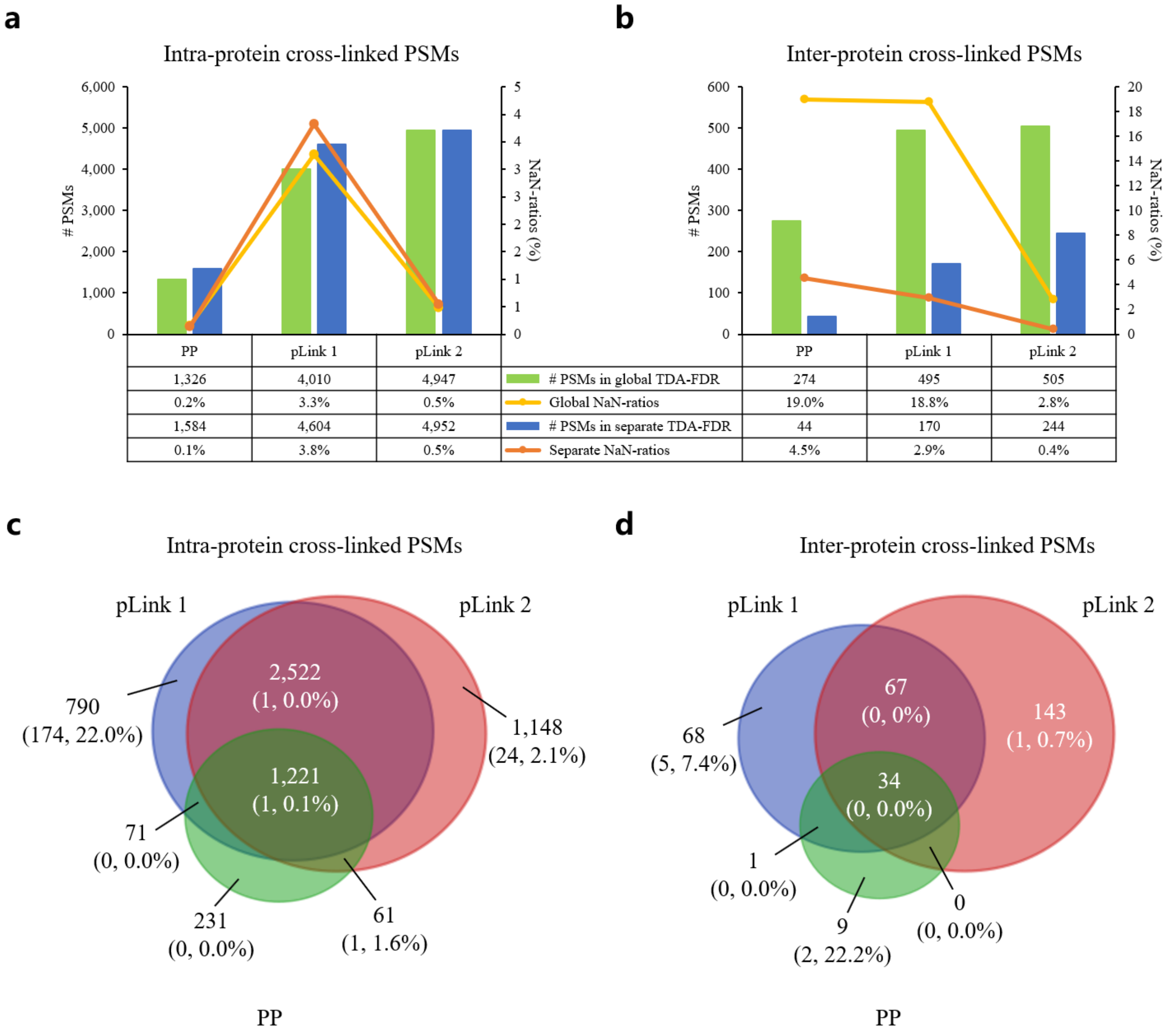

Supplementary Figure 8. Compare pLink 2 with Protein Prospector (PP) and pLink 1 under different FDR controls on the E.coli-Leiker-^{15}N dataset. **a)** For intra-protein PSMs, more results were reported by each search engine under separate FDR control, and its percentage of NaN-ratios was slightly higher than that under global FDR control. **b)** For inter-protein PSMs, many fewer results were reported under separate FDR control, and its percentage of NaN-ratios decreased, especially for Protein Prospector and pLink 1. And even if under the same strategy of separate FDR control, Protein Prospector reported many fewer **c)** intra-protein PSMs and **d)** inter-protein PSMs, and the percentage of NaN-ratios of inter-protein PSMs unique to Protein Prospector was much higher than those of pLink 1 and pLink 2.

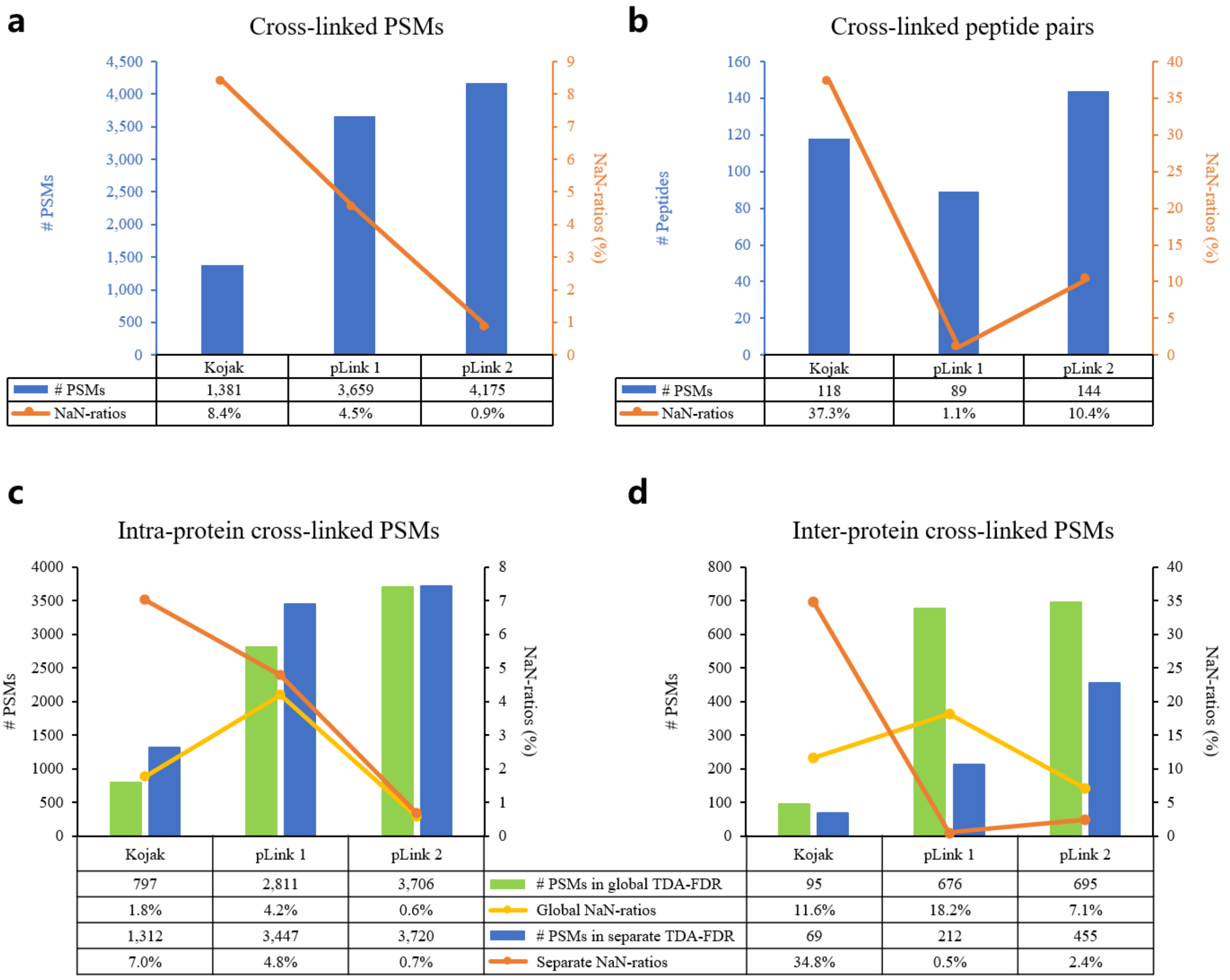

Supplementary Figure 9. Performance evaluation on the E.coli-SS-^{15}N dataset. **a)** Analyses of the identified cross-linked PSMs. **b)** Analyses of the identified cross-linked peptide pairs. The histograms denote the total numbers of a) PSMs or b) peptide pairs identified by each search engine under separate FDR control of intra-protein and inter-protein results, and the curves denote the percentage of NaN-ratio a) PSMs or b) peptide pairs in the corresponding histograms. As the number of disulfide-linked peptides identified by pLink 1 was small, and only one peptide pair could not find the corresponding ^{15}N-labeled precursor ion, making the percentage of NaN-ratios at peptide pair level less statistically significant, which was abnormally less than that at PSM level. **c)** For intra-protein PSMs, more results were reported under separate FDR control, and its percentage of NaN-ratios was slightly higher than that under global FDR control. **d)** For inter-protein PSMs, many fewer results were reported under separate FDR control, and its percentage of NaN-ratios decreased, especially for pLink 1.

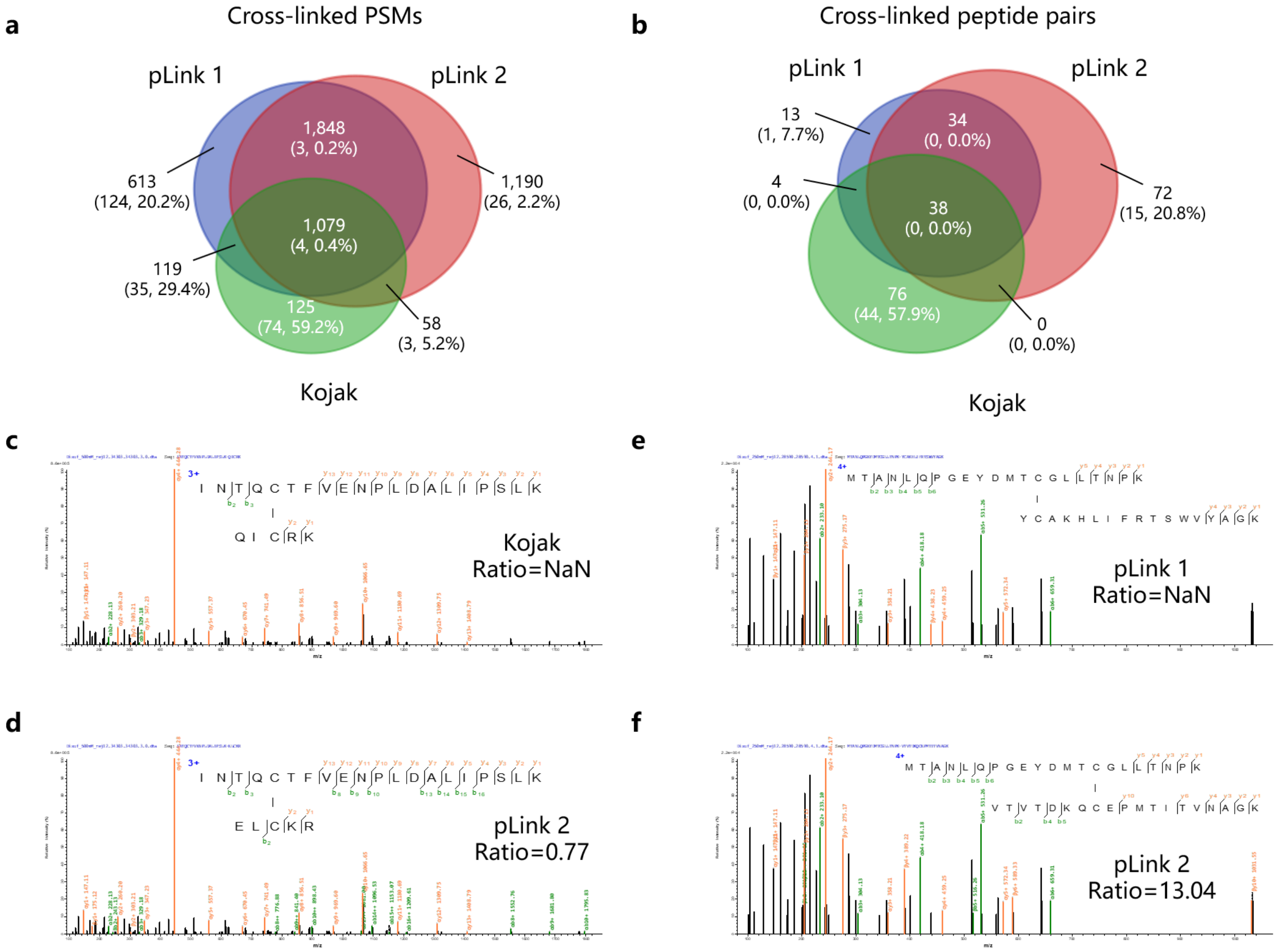

Supplementary Figure 10. Analysis of NaN-ratio results on the E.coli-SS-^{15}N dataset. **a)** PSMs uniquely identified by pLink 2 had the lowest percentage of NaN-ratios. Numbers in parentheses indicate the number and the percentage of NaN-ratio results that lie in the corresponding region. For example, 613 (124, 20.2%) means that pLink 1 uniquely identified 613 cross-linked PSMs, of which 124 were NaN-ratios, accounting for 20.2% of 613. **b)** The numbers of disulfide-linked peptides identified by three search engines were too small compared with those on the E.coli-Leiker-^{15}N dataset, making the percentage of NaN-ratios in peptide level less statistically significant. An example of **c)** NaN-ratio peptide pair identified by Kojak and **d)** *non*-NaN-ratio peptide pair identified by pLink 2 for the same spectrum. An example of **e)** NaN-ratio peptide pair identified by pLink 1 and **f)** *non*-NaN-ratio peptide pair identified by pLink 2 for the same spectrum. A probable reason for the high percentage of NaN-ratios of Kojak and pLink 1 might be that they could not recall correct β-peptides in top-*k*.

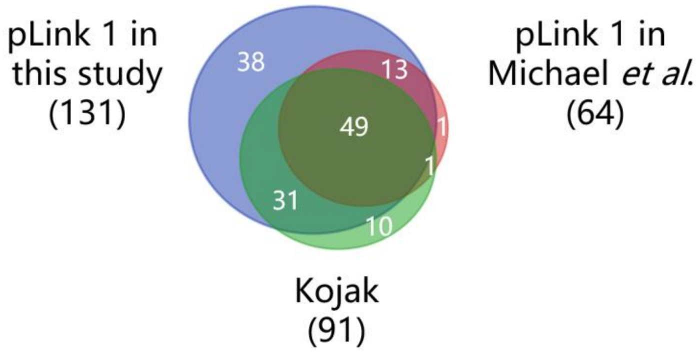

Supplementary Figure 11. On the SCF(FBXL3)-BS3 dataset, comparison of the number of cross-linked residue pairs identified by pLink 1 between this study and the publication of Kojak[7]. This study used one large precursor tolerance of ±5Da when searching, and five small precursor tolerance windows of ±10ppm when filtering, which is commonly used by pLink 1 users, whereas the previous publication of Kojak[7] used only one small precursor tolerance of ±15ppm when searching, which led to many fewer results. If one large precursor tolerance was used, pLink 1 identified 131 cross-linked residue pairs, which were more than that of Kojak and covered ~88% of the latter.

a

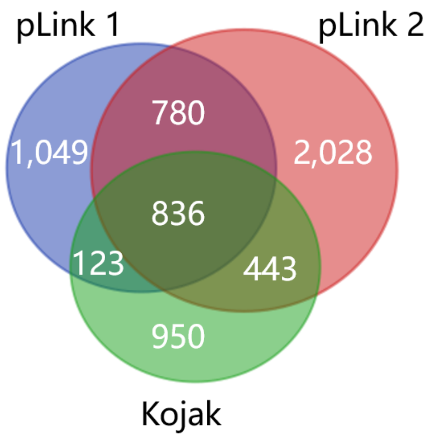

b

Sensitivity	+ *E. coli*	+ Worm	+ Human
Kojak	88.4%	66.5%	66.5%
pLink 1	98.3%	89.8%	88.5%
pLink 2	98.3%	96.8%	97.0%
**Precision**	**+ *E. coli***	**+ Worm**	**+ Human**
Kojak	92.8%	82.0%	88.0%
pLink 1	99.6%	98.9%	98.8%
pLink 2	99.3%	99.0%	98.7%

Supplementary Figure 12. Performance evaluation on the Ca_V1.1-SS dataset. **a**) A real-world protein complex sample was searched using Kojak, pLink 1, and pLink 2. A total of 836 cross-linked PSMs were identified consistently by the three engines; these were used to be a new and fair standard dataset. **b)** The sensitivities and precisions of the three engines. "+ *E. coli*" database contains sequences from 8 target proteins and the *E. coli* whole proteome database, and "+ Worm" and "+ Human" are similar to "+ *E. coli*".

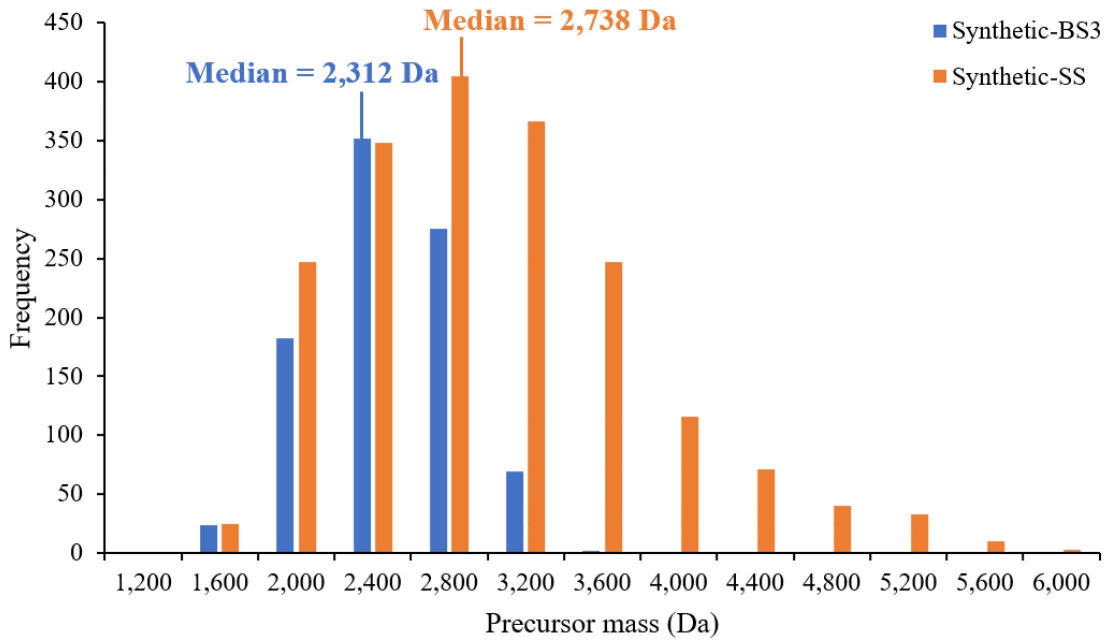

Supplementary Figure 13. Distribution of precursor masses on the Synthetic-BS3 and the Synthetic-SS datasets. The bin size is 400 Da, and a majority of the precursor masses on the Synthetic-SS dataset are larger than those on the Synthetic-BS3 dataset.

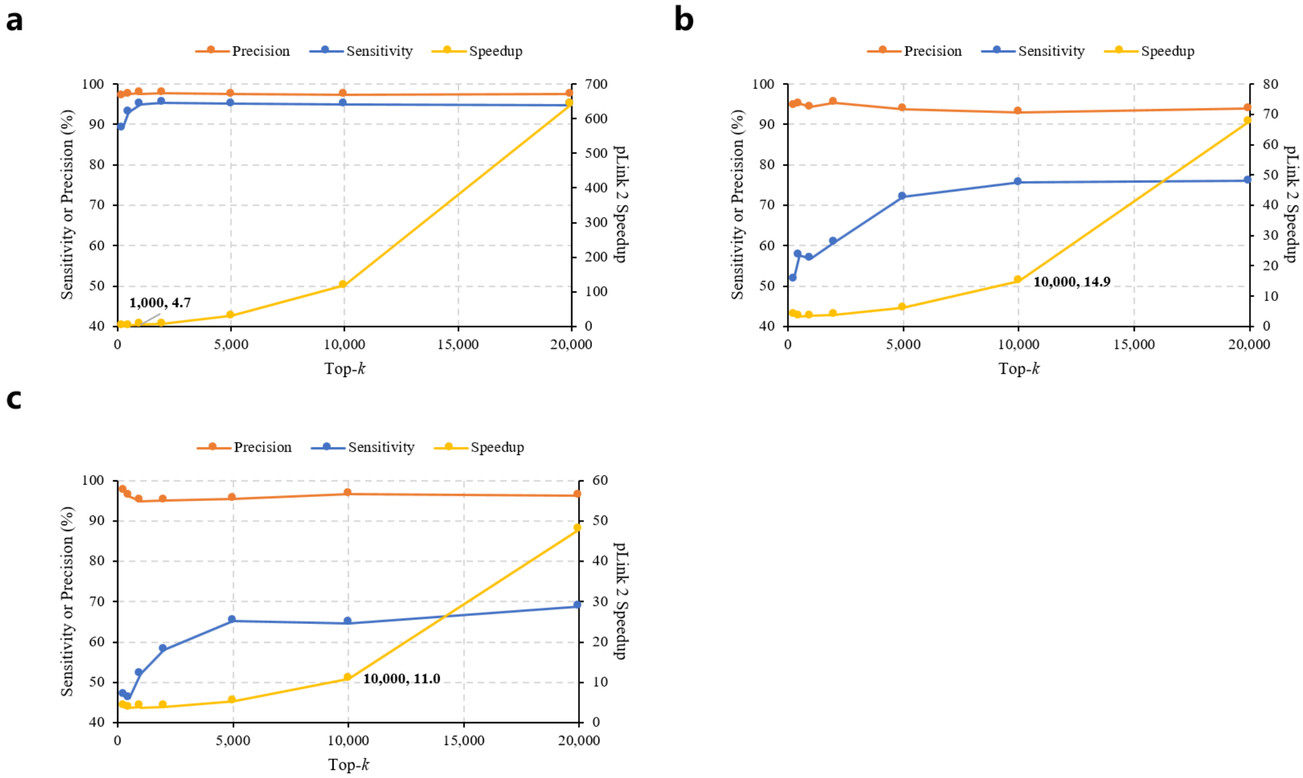

Supplementary Figure 14. The speedup of pLink 2 on the Synthetic-SS dataset. **a)** pLink 2 achieved a 4.7 times speedup when searching against the *E. coli* entrapment database. The horizontal axis is the number of top-k scored single peptides kept in Kojak, starting from its default value of 250. Speedup was measured when the sensitivity of Kojak remained steady. **b-c)** Similar to a), but against b) the worm and c) the human entrapment database, respectively.

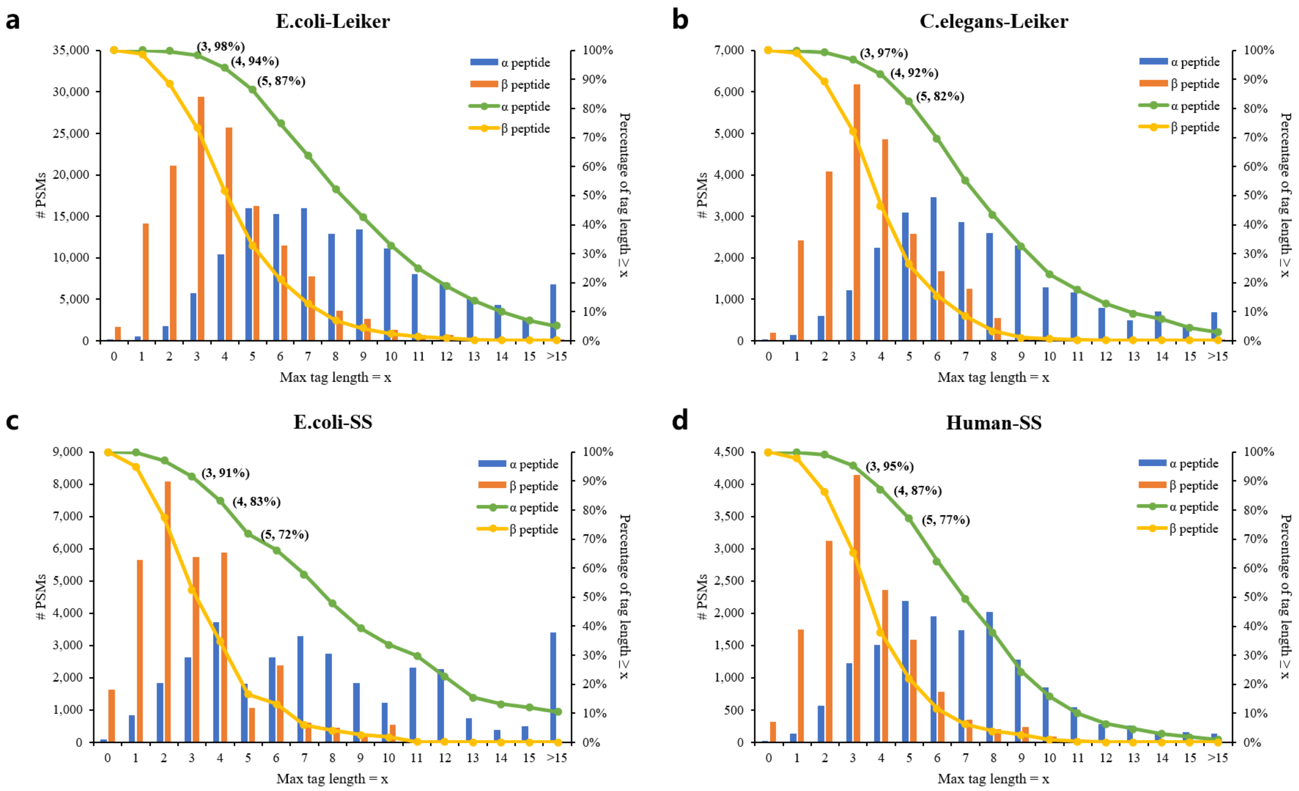

Supplementary Figure 15. The distribution (bar) and the accumulated percentage (line) of max length of extracted tag in α- and β-peptide from the identified cross-linked PSMs on four previously published datasets of **a)** E.coli-Leiker, **b)** C.elegans-Leiker, **c)** E.coli-SS, and **d)** Human-SS. For example, on E.coli-Leiker dataset, at least one 5-tag could be extracted in 87% of the identified PSMs.

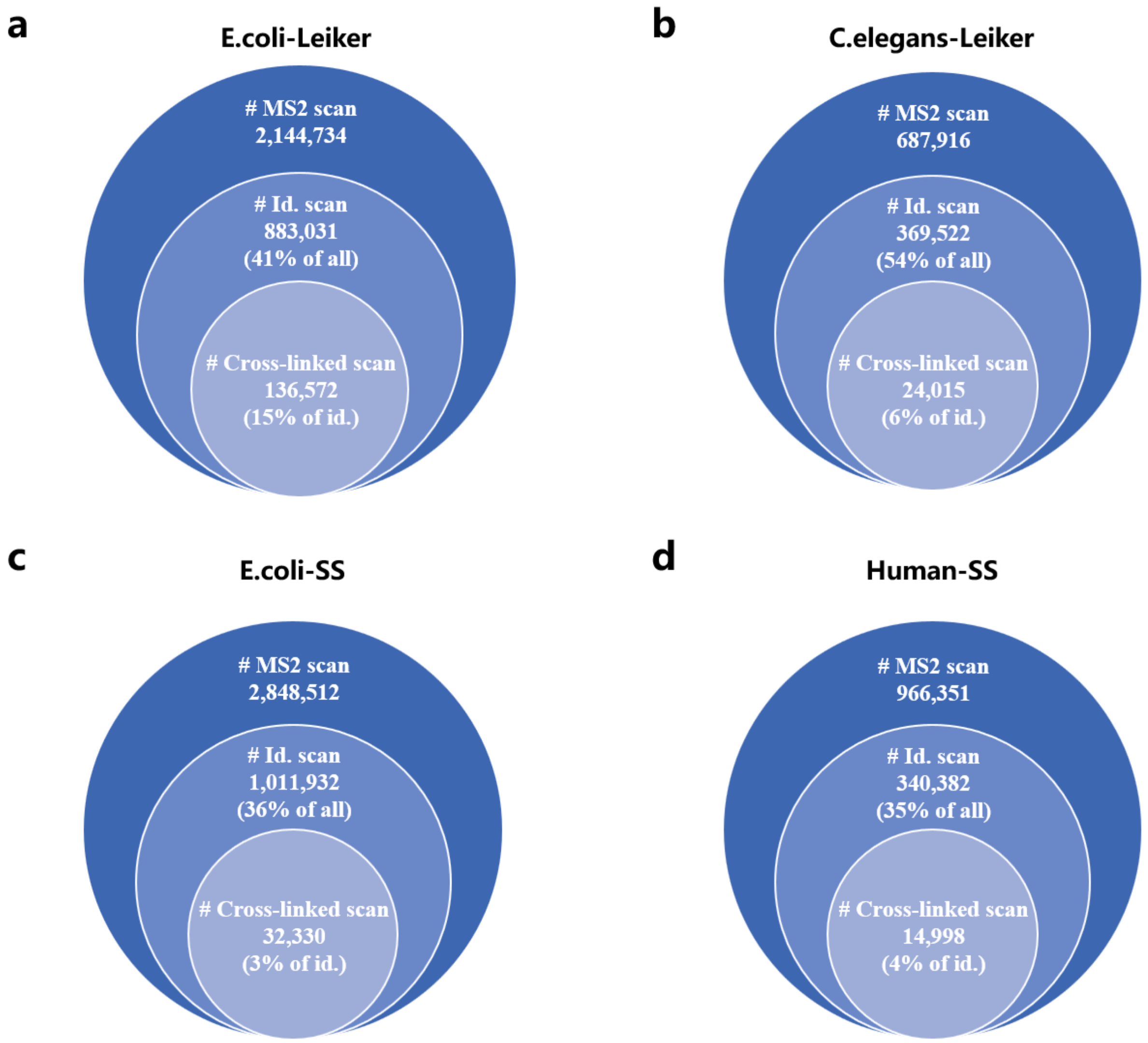

Supplementary Figure 16. The identification rate and the proportion of identified cross-linked scans in all identified scans on four previously published datasets of **a)** E.coli-Leiker, **b)** C.elegans-Leiker, **c)** E.coli-SS, and **d)** Human-SS.

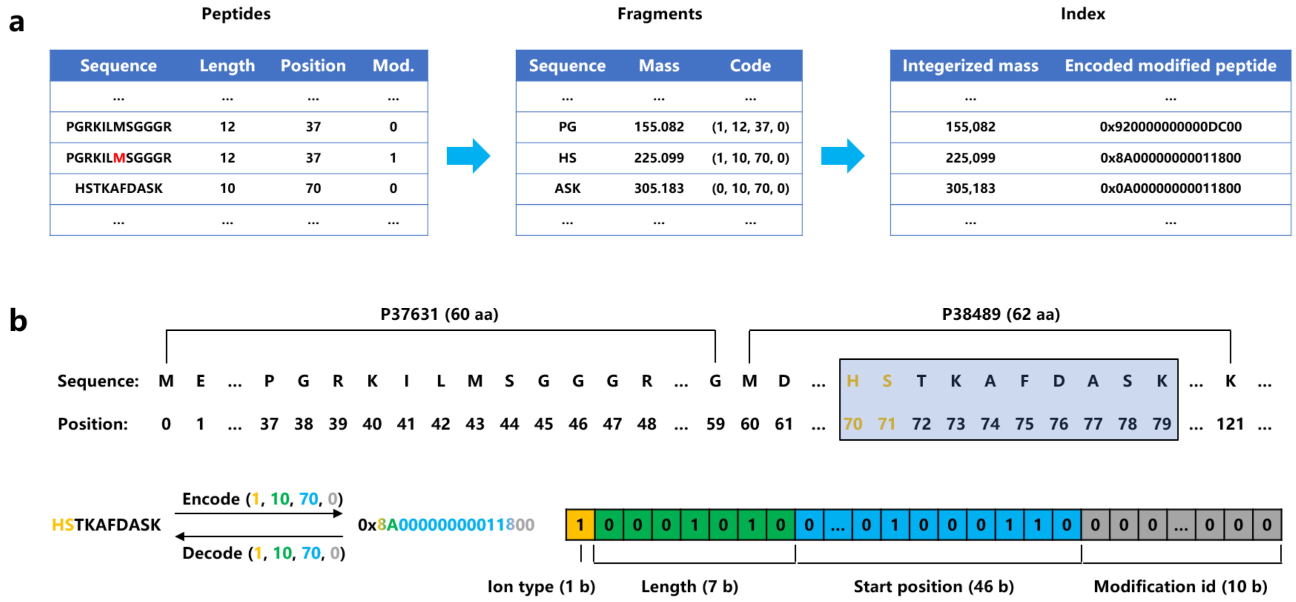

Supplementary Figure 17. The scheme of constructing a fragment index. **a)** The workflow of constructing a fragment index for all modified peptides. First, each modified peptide was represented by its length (l), start position (p) in the sequence concatenating all proteins in the database, and the modification identifier (m) among all modified peptides from the peptide sequence. Next, for each modified peptide, all neutral masses of b, y fragment ions were generated. The ion type information (t) and the modified peptide information make up a tetrad code (t, l, p, m). Finally, each tetrad code was encoded into a 64-bit integer. **b)** An example of encoding and decoding a fragment ion. A fragment ion was represented by a tetrad code (t, l, p, m), which used 1 bit, 7 bits, 46 bits, and 10 bits to encode its ion type information, length, start position, and modification identifier respectively.

Supplementary Note 1. Evaluate the performance of ten search engines using synthetic datasets

In the main text, according to the evaluation result on the simulated datasets, only the top-3 highest-sensitivity search engines, namely pLink 2, pLink 1, and Kojak, were selected for further evaluation using synthetic datasets. In this supplementary note, we tested all ten search engines on the synthetic datasets.

Take the Synthetic-BS3 dataset as an example. First of all, ten search engines searched all 2,077 spectra against the original database. As the sensitivities of ten search engines varied greatly from one another, the intersection of identifications from all ten search engines would be very small (Original database in Supplementary Fig. 18). We thus only took the intersection of identifications from Kojak, pLink 1, and pLink 2 as a new and fair standard dataset for searching against increasingly larger databases generated by appending the *E. coli*, worm, or human database as an entrapment to the original database. The new and fair Synthetic-BS3 dataset consists of 904 PSMs (same as in Fig. 2a).

Supplementary Fig. 18 shows that only six of ten search engines successfully finished searching against the *E. coli* entrapment database, they were Xolik, MetaMorpheusXL, Kojak, PP, pLink 1, and pLink 2 sorted by sensitivity in ascending order. Furthermore, the sensitivities of search engines decreased as the database size increased (Supplementary Fig. 19a), while the precisions were relatively stable (Supplementary Fig. 19b). Evaluations using three different entrapment databases showed that pLink 2 achieved the highest sensitivity, followed closely by pLink 1. In contrast, the sensitivities of PP, Kojak, MetaMorpheusXL, and Xolik decreased significantly, especially for MetaMorpheusXL and Xolik, whose sensitivities decreased down to less than 10% with both the worm and the human entrapment databases. The results obtained for the Synthetic-SS dataset were similar to those obtained for the Synthetic-BS3 dataset, except that MetaMorpheusXL and Xolik both threw an “OutOfMemoryError” exception and failed to finish searching against the worm and the human entrapment databases (Supplementary Figs. 20 and 21).

Search engines were also compared in terms of computing time. Where possible, 8 threads were used for each search engine. The normalized computing times for ten search engines (Windows Server, Intel Xeon E5-2670 CPU with 32 cores, 2.6 GHz, 128 GB RAM) are shown in Supplementary Table 8. Most search engines did not finish searching against the worm and the human entrapment databases. pLink 2 was the fastest one that passed all evaluations and achieved the highest sensitivities. Although Xolik was faster than pLink 2, it had very low sensitivity (Supplementary Fig. 18) and failed to control the memory usage when searching the disulfide bond data against big databases (Supplementary Fig. 20).

To the best of our knowledge, xQuest, Xilmass, StavroX, and Xi have not been shown to support cross-linked peptide identification against the *full human proteome* database, while the other six do. Some search engines in the former group may be used for proteome-wide searches assisted by additional techniques. For example, xQuest used an isotopically labeled cross-linker to search against the full *E. coli* proteome[1], and Xi used a restricted database based on standard bottom-up proteomics to search against the human proteome[9]. In contrast, search engines in the latter group can be used for proteome-wide searches without additional techniques. They can be further categorized into three types according to sensitivity: Xolik and MetaMorpheusXL have very low sensitivities; PP and Kojak have moderately higher sensitivities; pLink 1 and pLink 2 have the highest sensitivities. Performance comparison among all ten search engines using synthetic datasets shows that pLink 2, pLink 1, and Kojak were indeed the top-3 highest-sensitivity search engines, which confirmed the conclusion drawn from the evaluation using simulated datasets.

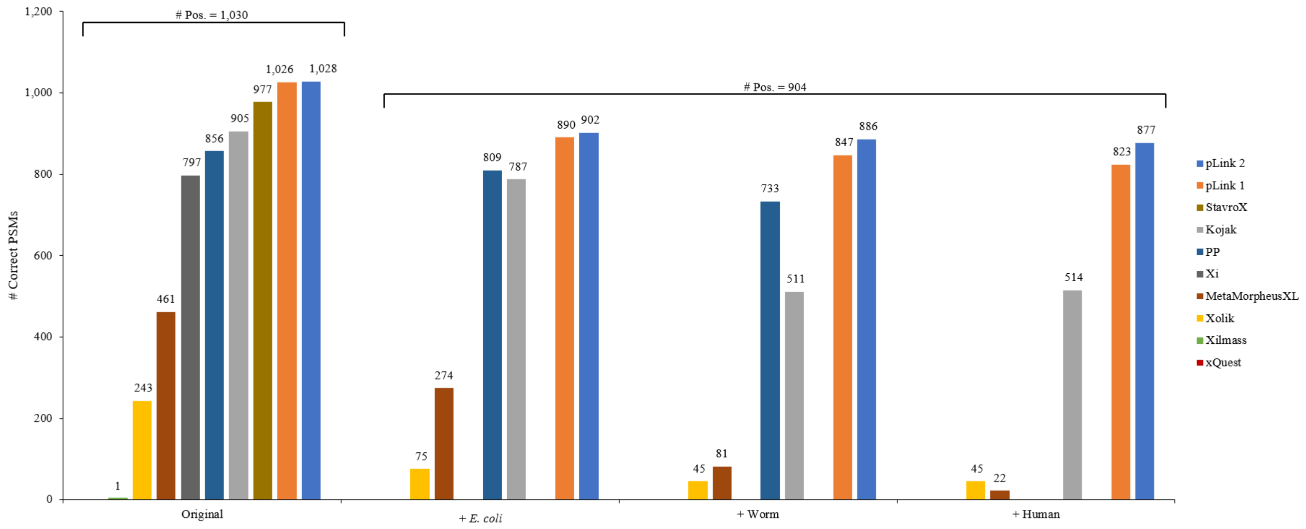

Supplementary Figure 18. The numbers of correctly identified PSMs on the Synthetic-BS3 dataset. The dataset contains 904 spectra and the "Original" database contains only the sequences of 38 synthetic peptides, "+ *E. coli*" database contains sequences from the "Original" database and the *E. coli* whole proteome database, and "+ Worm" and "+ Human" are similar to "+ *E. coli*".

a

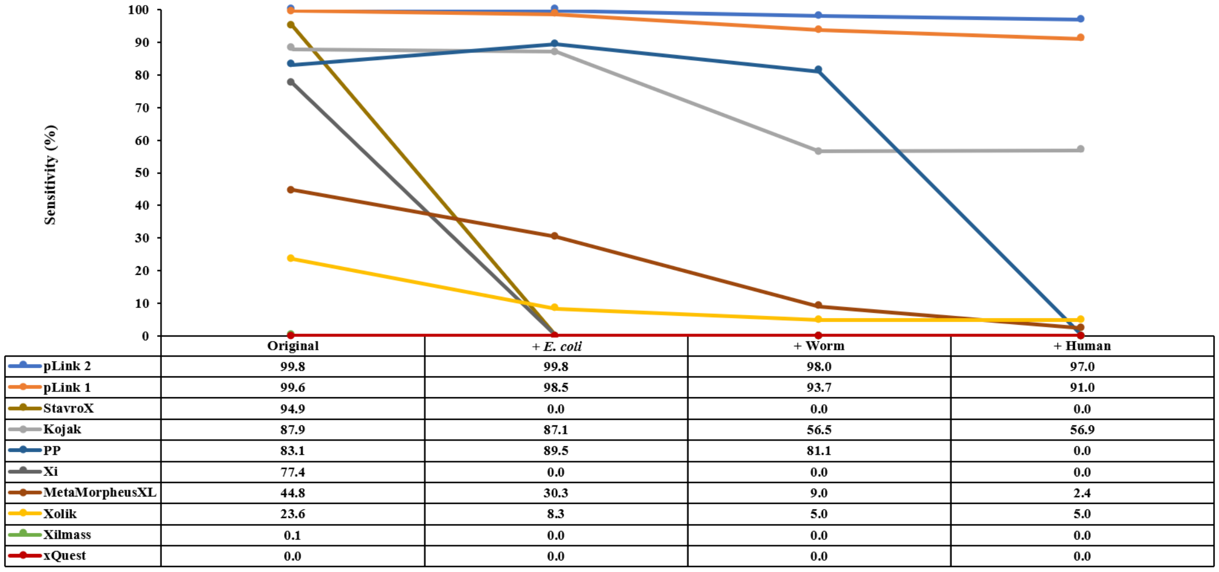

	Original	+ *E. coli*	+ Worm	+ Human
pLink 2	99.8	99.8	98.0	97.0
pLink 1	99.6	98.5	93.7	91.0
StavroX	94.9	0.0	0.0	0.0
Kojak	87.9	87.1	56.5	56.9
PP	83.1	89.5	81.1	0.0
Xi	77.4	0.0	0.0	0.0
MetaMorpheusXL	44.8	30.3	9.0	2.4
Xolik	23.6	8.3	5.0	5.0
Xilmass	0.1	0.0	0.0	0.0
xQuest	0.0	0.0	0.0	0.0

b

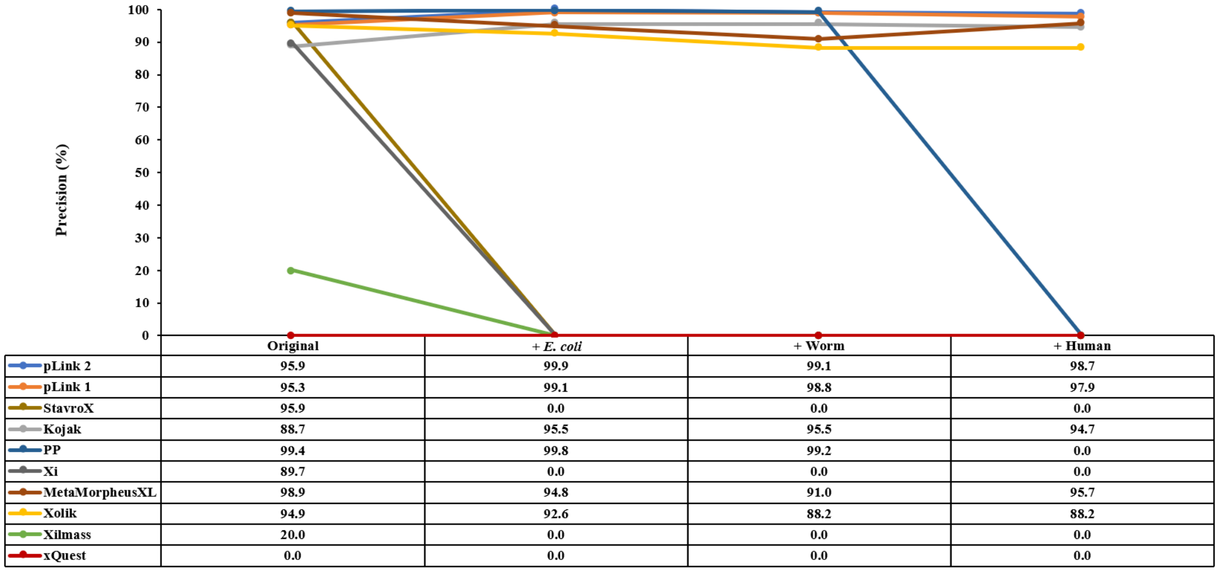

	Original	+ *E. coli*	+ Worm	+ Human
pLink 2	95.9	99.9	99.1	98.7
pLink 1	95.3	99.1	98.8	97.9
StavroX	95.9	0.0	0.0	0.0
Kojak	88.7	95.5	95.5	94.7
PP	99.4	99.8	99.2	0.0
Xi	89.7	0.0	0.0	0.0
MetaMorpheusXL	98.9	94.8	91.0	95.7
Xolik	94.9	92.6	88.2	88.2
Xilmass	20.0	0.0	0.0	0.0
xQuest	0.0	0.0	0.0	0.0

Supplementary Figure 19. Performance evaluation on the Synthetic-BS3 dataset. The **a)** sensitivity and **b)** precision of ten search engines when searching against original database plus entrapment databases of different sizes. Search engines were sorted by sensitivity in original database in descending order.

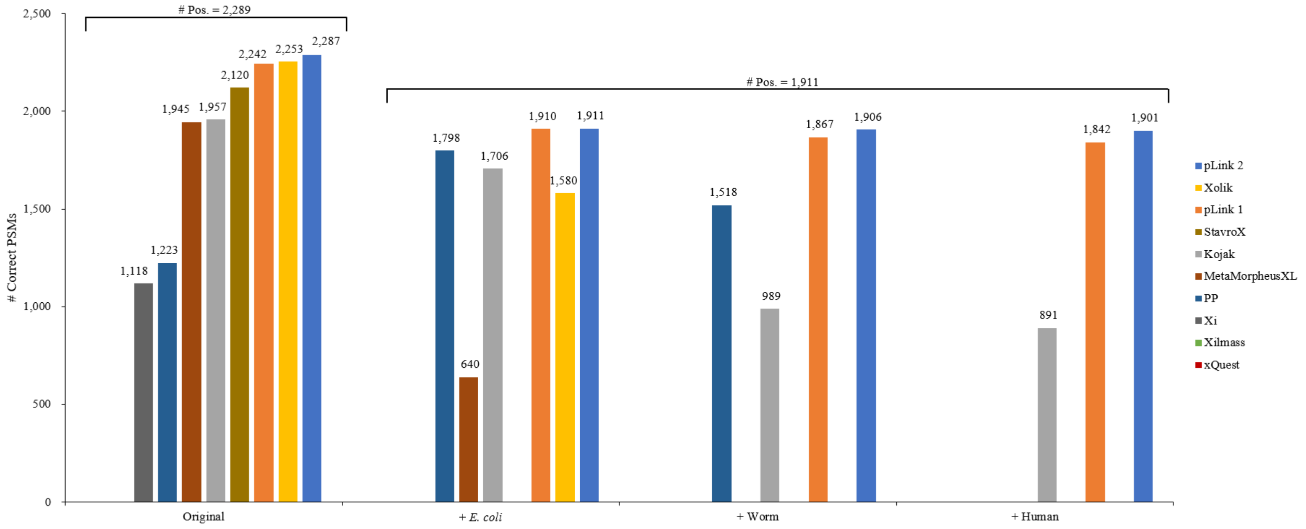

Supplementary Figure 20. The numbers of correctly identified PSMs on the Synthetic-SS dataset. The dataset contains 1,911 spectra and the "Original" database contains only the sequences of 72 synthetic peptides, "+ *E. coli*" database contains sequences from the "Original" database and the *E. coli* whole proteome database, and "+ Worm" and "+ Human" are similar to "+ *E. coli*".

a

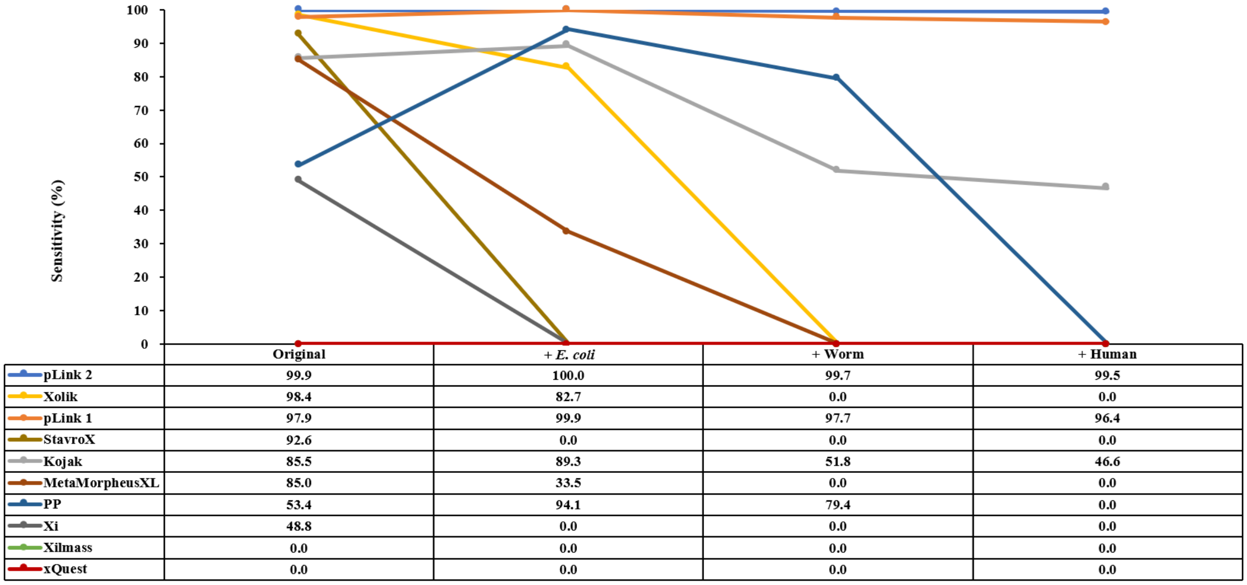

	Original	+ *E. coli*	+ Worm	+ Human
pLink 2	99.9	100.0	99.7	99.5
Xolik	98.4	82.7	0.0	0.0
pLink 1	97.9	99.9	97.7	96.4
StavroX	92.6	0.0	0.0	0.0
Kojak	85.5	89.3	51.8	46.6
MetaMorpheusXL	85.0	33.5	0.0	0.0
PP	53.4	94.1	79.4	0.0
Xi	48.8	0.0	0.0	0.0
Xilmass	0.0	0.0	0.0	0.0
xQuest	0.0	0.0	0.0	0.0

b

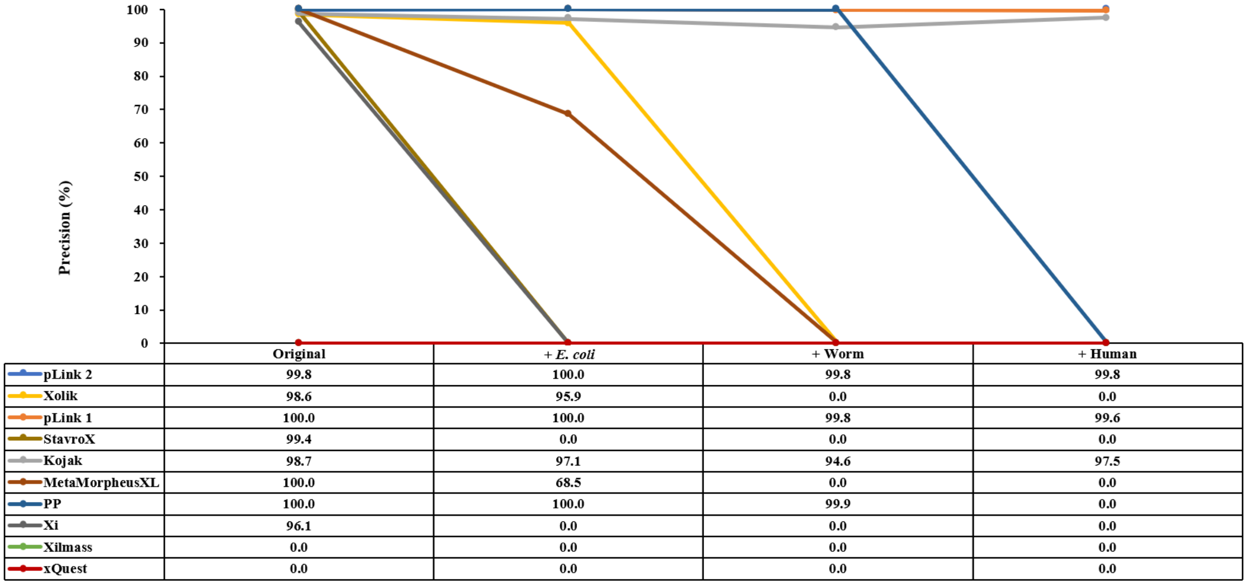

	Original	+ *E. coli*	+ Worm	+ Human
pLink 2	99.8	100.0	99.8	99.8
Xolik	98.6	95.9	0.0	0.0
pLink 1	100.0	100.0	99.8	99.6
StavroX	99.4	0.0	0.0	0.0
Kojak	98.7	97.1	94.6	97.5
MetaMorpheusXL	100.0	68.5	0.0	0.0
PP	100.0	100.0	99.9	0.0
Xi	96.1	0.0	0.0	0.0
Xilmass	0.0	0.0	0.0	0.0
xQuest	0.0	0.0	0.0	0.0

Supplementary Figure 21. Performance evaluation on the Synthetic-SS dataset. The **a)** sensitivity and **b)** precision of ten search engines when searching against original database plus entrapment databases of different sizes. Search engines were sorted by sensitivity in original database in descending order.

Supplementary Table 8. The normalized computing times of ten search engines on two synthetic datasets

Dataset		xQuest	Xilmass	Xolik	MetaMorpheusXL	Xi	PP	Kojak	StavroX	pLink 1	pLink 2	Real time[g]
Synthetic-BS3	Original	**b**	5.5	1.8	2.0	56.8	26.0	2.3	1.8	35.5	1.0	0.1
	+ *E. coli*		**c**	0.5	5.1	**c**	7.7	1.7	**f**	45.4	1.0	0.5
	+ Worm			0.3	35.2		11.6	2.4		50.8	1.0	3.5
	+ Human			0.2	56.7		**e**	2.1		38.3	1.0	6.4
Synthetic-SS	Original		**d**	2.0	1.2	4.7	35.5	2.6	3.1	32.7	1.0	0.2
	+ *E. coli*			0.6	5.9	**c**	17.0	3.3	**f**	60.2	1.0	1.2
	+ Worm			**c**	**c**		10.4	3.8		64.3	1.0	17.7
	+ Human						**e**	4.0		69.7	1.0	31.9
Average[a]		-	-	0.9	17.7	-	18.0	2.8	-	49.6	1.0	-

a The average of normalized computing times was calculated only for search engines that passed no fewer than six tests.

b xQuest threw an exception "Illegal division by zero at /home/xqxp/xquest/V2_1_1/xquest/bin/compare_peaks3.pl line 2246" and did not report any results.

c These search engines threw an exception "OutOfMemoryError" and did not report any results.

d Xilmass did not support the disulfide bond cross-linker and could not set user defined cross-linkers.

e PP threw a "File open failure" exception and failed to start searching.

f StavroX did not finish searching within one week.

g The real search times for pLink 2 are shown in minutes.

Supplementary Note 2. The relationship between the global FDR and the subgroup FDRs of intra-protein and inter-protein cross-linked identifications

The early study estimated a global FDR for all cross-linked peptide pair identifications[4]. That is, the identifications of intra-protein and inter-protein peptide pairs were subject to FDR estimation in combination. Later, some studies noted the high actual FDR of inter-protein peptide pairs and therefore proposed separate FDR controls for intra-protein and inter-protein peptide pairs[2,6]. In fact, the relationship between intra-protein and inter-protein peptide pairs is quite similar to the relationship between unmodified and modified peptides, which had been formally studied in the work of Fu[16]. Here, we investigate the subgroup FDRs of intra-protein and inter-protein peptide pairs under the global FDR control by following the same framework of Fu's work.

Let subscript $k \in \{intra, inter\}$ denote the subgroup of intra-protein (*intra*) and inter-protein (*inter*) peptide pair identifications, respectively. Then following Fu's formalization, we define:

(i) T, a true peptide pair identification;

(ii) F, a false peptide pair identification;

(iii) I_k, a peptide pair identification belonging to subgroup k;

(iv) FDR(x), the global FDR of all cross-linked peptide pair identifications with scores greater than x (assume the greater the better), where both intra-protein and inter-protein peptide pairs are included;

(v) $\mathrm{FDR}_k(x)$, the subgroup FDR of type k identifications, *e.g.* $\mathrm{FDR}_{intra}(x)$ for intra-protein identifications and $\mathrm{FDR}_{inter}(x)$ for inter-protein identifications.

According to definitions, $\mathrm{FDR}(x) = P(F \mid X > x)$ and $\mathrm{FDR}_k(x) = P(F \mid I_k, X > x)$. Following the derivation by Fu[16], $\mathrm{FDR}_k(x)$ can be written as

$$\begin{aligned}
&\mathrm{FDR}_k(x) \\
&= P(F|I_k, X > x) \\
&= \frac{P(F, I_k|X > x)}{P(I_k|X > x)} \\
&= \frac{P(I_k|F, X > x)P(F|X > x)}{P(I_k|F, X > x)P(F|X > x) + P(I_k|T, X > x)P(T|X > x)} \\
&= \frac{P(F|X > x)}{P(F|X > x) + \frac{P(I_k|T, X > x)}{P(I_k|F, X > x)}\big(1 - P(F|X > x)\big)} \\
&= \frac{\mathrm{FDR}(x)}{\mathrm{FDR}(x) + \frac{P(I_k|T, X > x)}{P(I_k|F, X > x)}(1 - \mathrm{FDR}(x))},
\end{aligned} \tag{1}$$

where $P(I_k \mid T, X > x)$ is the probability that an identification belongs to subgroup k given that this identification is true and scores better than x, and $P(I_k \mid F, X > x)$ is the probability that an identification belongs to subgroup k given that this identification is false and scores better than x. From an experimental perspective it is much more likely to observe intra-protein cross-links, so $P(I_{intra} \mid T, X > x) > P(I_{inter} \mid T, X > x)$. From a bioinformatic perspective it is much more likely to randomly match inter-protein cross-links since the search space of inter-protein cross-links is far more larger than that of intra-protein cross-links, so $P(I_{intra} \mid F, X > x) < P(I_{inter} \mid F, X > x)$. Therefore, we have $\frac{P(I_{intra}|T,X>x)}{P(I_{intra}|F,X>x)} > \frac{P(I_{inter}|T,X>x)}{P(I_{inter}|F,X>x)}$ and by Supplementary Equation 1, $\mathrm{FDR}_{intra} < \mathrm{FDR}_{inter}$.

Additionally, the relationship between the global FDR of all cross-linked identifications and subgroup FDRs of intra-protein and inter-protein identifications can be deduced by the following equation:

$$
\begin{aligned}
&\mathrm{FDR}(x) \\
&= P(F|X > x) \\
&= \frac{P(F, X > x)}{P(X > x)} \\
&= \frac{P(F, I_{intra}, X > x) + P(F, I_{inter}, X > x)}{P(X > x)} \\
&= \frac{P(F|I_{intra}, X > x) \cdot P(I_{intra}, X > x) + P(F|I_{inter}, X > x) \cdot P(I_{inter}, X > x)}{P(X > x)} \\
&= P(F|I_{intra}, X > x) \cdot \frac{P(I_{intra}, X > x)}{P(X > x)} + P(F|I_{inter}, X > x) \cdot \frac{P(I_{inter}, X > x)}{P(X > x)} \\
&= \mathrm{FDR}_{intra}(x) \cdot P(I_{intra}|X > x) + \mathrm{FDR}_{inter}(x) \cdot P(I_{inter}|X > x).
\end{aligned} \tag{2}
$$

Therefore, the global FDR of all cross-linked identifications is a linear combination of subgroup FDRs of intra-protein and inter-protein identifications, with positive weights of $P(I_{intra} \mid X > x)$ and $P(I_{inter} \mid X > x)$. Thus, FDR(x) lies between FDR_{intra} and FDR_{inter}. Since in general $\mathrm{FDR}_{intra} < \mathrm{FDR}_{inter}$, we have

$$
\mathrm{FDR}_{intra}(x) < \mathrm{FDR}(x) < \mathrm{FDR}_{inter}(x). \tag{3}
$$

This explains why the global FDR control often results in a higher subgroup FDR of inter-protein identifications. If intra-protein and inter-protein identifications are separately filtered to achieve the same FDR level as the global FDR(x), then the score threshold for intra-protein x_{intra} has to be decreased from x and the score threshold for inter-protein x_{inter} has to be increased from x, which leads to more intra-protein results and fewer inter-protein results. Fig. 3 in the main text and Supplementary Fig. 9 show this phenomenon.

Supplementary Note 3. Deducing an NaN-FDR for a search engine independent of the TDA-FDR

Suppose N target PSMs are reported by a search engine, containing T correct PSMs and F incorrect PSMs, then we have the equation

$$N = T + F. \tag{4}$$

After checking the quantification ratios of these N PSMs by pQuant[17], suppose there are M NaN-ratio PSMs, then some come from the correct PSMs, and the other come from the incorrect PSMs. Following Zhou's formulation[18], let e_1 denotes the percentage of NaN-ratio PSMs in correct PSMs, which is called the false positive rate of ^{15}N metabolic labeling evaluation; let e_2 denotes the percentage of *non*-NaN-ratio PSMs in incorrect PSMs, which is called the false negative rate of ^{15}N metabolic labeling evaluation. Therefore, we have another equation

$$M = T * e_1 + F * (1 - e_2). \tag{5}$$

By solving Supplementary Equation 4 and Supplementary Equation 5 simultaneously, the number of incorrect PSMs F can be obtained using the following formula:

$$F = \frac{M - N * e_1}{1 - e_1 - e_2}. \tag{6}$$

Then the NaN-FDR of the N target PSMs is F / N, which can be written as

$$\text{NaN-FDR} = \frac{M/N - e_1}{1 - e_1 - e_2}, \tag{7}$$

where M / N is the percentage of NaN-ratio results. Therefore, given e_1 and e_2, the NaN-FDR has a linear correlation with the percentage of NaN-ratios, and a smaller percentage of NaN-ratio results indicates a lower NaN-FDR.

Estimation of NaN-FDR is based on estimations of e_1 and e_2. The false positive rate e_1 is estimated by the percentage of NaN-ratio PSMs in the intersection of the results of different search engines, because a PSM is more likely to be correct if it is consistently reported by multiple search engines[19-21]. Actually, estimated on the E.coli-Leiker-^{15}N dataset and the E.coli-SS-^{15}N dataset, e_1 is almost zero (Supplementary Figs. 22 and 23). The false negative rate e_2 is estimated by the percentage of *non*-NaN-ratio PSMs in decoy matches which passed the TDA-FDR control, because a decoy PSM is always considered as incorrect. Estimated on the E.coli-Leiker-^{15}N dataset and the E.coli-SS-^{15}N dataset, e_2 varied from 30% to 80% (Supplementary Figs. 22 and 23).

a

Cross-linked PSMs	Kojak	pLink 1	pLink 2
# PSMs	2,672	4,774	5,196
NaN-ratios (%)	6.4	3.8	0.5
e_1 (%)	0.0	0.0	0.0
e_2 (%)	34.5	50.3	77.4
NaN-FDR (%)	9.8	7.6	2.4

b

Cross-linked peptide pairs	Kojak	pLink 1	pLink 2
# Peptides	467	558	541
NaN-ratios (%)	16.9	5.4	1.3
e_1 (%)	0.0	0.0	0.0
e_2 (%)	38.2	48.9	72.3
NaN-FDR (%)	27.4	10.5	4.7

c

Intra-protein cross-links	Kojak	pLink 1	pLink 2
# PSMs in global TDA-FDR	1,088	4,010	4,947
NaN-FDR (%)	8.1	5.0	0.7
# PSMs in separate TDA-FDR	2,620	4,604	4,952
NaN-FDR (%)	9.1	7.7	2.4

d

Inter-protein cross-links	Kojak	pLink 1	pLink 2
# PSMs in global TDA-FDR	81	495	505
NaN-FDR (%)	24.5	37.8	12.3
# PSMs in separate TDA-FDR	52	170	244
NaN-FDR (%)	44.1	5.9	1.8

Supplementary Figure 22. Analysis results based on the estimated NaN-FDR on the E.coli-Leiker-^{15}N dataset. pLink 2 identified the most cross-linked **a)** PSMs and **b)** peptides with the lowest percentage of NaN-ratios and NaN-FDR. **c)** For intra-protein PSMs, more results were reported under separate FDR control, and its NaN-FDR was slightly higher than that under global FDR control. **d)** For inter-protein PSMs, many fewer results were reported under separate FDR control, and its NaN-FDR decreased, especially for pLink 1.

a

Cross-linked PSMs	Kojak	pLink 1	pLink 2
# PSMs	1,381	3,659	4,175
NaN-ratios (%)	8.4	4.5	0.9
e_1 (%)	0.4	0.4	0.4
e_2 (%)	43.4	53.8	48.1
NaN-FDR (%)	14.3	9.1	1.0

b

Cross-linked peptide pairs	Kojak	pLink 1	pLink 2
# Peptides	118	89	144
NaN-ratios (%)	37.3	1.1	10.4
e_1 (%)	0.0	0.0	0.0
e_2 (%)	46.5	54.5	48.4
NaN-FDR (%)	69.7	2.5	20.2

c

Intra-protein cross-links	Kojak	pLink 1	pLink 2
# PSMs in global TDA-FDR	797	2,811	3,706
NaN-FDR (%)	2.5	8.4	0.4
# PSMs in separate TDA-FDR	1,312	3,447	3,720
NaN-FDR (%)	11.8	9.6	0.6

d

Inter-protein cross-links	Kojak	pLink 1	pLink 2
# PSMs in global TDA-FDR	95	676	695
NaN-FDR (%)	19.9	38.9	13.0
# PSMs in separate TDA-FDR	69	212	455
NaN-FDR (%)	61.2	0.2	4.0

Supplementary Figure 23. Analysis results based on the estimated NaN-FDR on the E.coli-SS-^{15}N dataset. **a)** pLink 2 identified the most disulfide-linked PSMs with the lowest percentage of NaN-ratios and NaN-FDR. **b)** As the number of disulfide-linked peptides identified by pLink 1 was small, and only one peptide pair could not find the corresponding ^{15}N-labeled precursor ion, making the estimated NaN-FDR at peptide level less statistically significant, which was abnormally less than that at PSM level. **c)** For intra-protein PSMs, more results were reported under separate FDR control, and its NaN-FDR was slightly higher than that under global FDR control. **d)** For inter-protein PSMs, many fewer results were reported under separate FDR control, and its NaN-FDR decreased, especially for pLink 1.

Supplementary Note 4. Evaluate the performance of XlinkX

The software XlinkX[22,23] is a search engine designed mainly for analysing data from MS-cleavable cross-linkers, but it also supports data analysis of non-cleavable cross-linkers. We have tried the latest XlinkX node in Proteome Discoverer 2.3 using the xlinkx23_noncleavable.pdAnalysis template file provided on the Heck lab's website (https://www.hecklab.com/software/xlinkx/). We found that XlinkX can analyse RAW files but cannot analyse MGF files, and therefore we cannot evaluate XlinkX using the simulated and synthetic datasets. We finally evaluated the performance of XlinkX using ^{15}N metabolically labeled datasets and entrapment databases, for which RAW files are available.

Credibility evaluation using ^{15}N metabolically labeled datasets

For the E.coli-Leiker-^{15}N dataset. As XlinkX only supports up to 1,500 proteins when analysing non-cleavable cross-linker data (https://www.hecklab.com/software/xlinkx/), the database for XlinkX search contained only 1,284 proteins identified from RAW files of the sample using a regular peptide search engine, pFind[24] (restricted search mode with parameters in Supplementary Table 5 except that the FDR was set as 5% at peptide level). The parameters for XlinkX search were the same as those for pLink 2 (Supplementary Table 5). pLink 2 also searched against the same restricted database of 1,284 proteins, so that results could be fairly compared.

XlinkX identified a total of 1,255 cross-linked PSMs with 1.4% NaN-ratios, while pLink 2 identified fourfold of that (5,450) with only 0.6% NaN-ratios. Furthermore, PSMs uniquely identified by pLink 2 had much lower percentage of NaN-ratios compared with PSMs uniquely identified by XlinkX (Supplementary Figs. 24a and 24b). Results at peptide pair level were similar to those at PSM level (Supplementary Figs. 24c and 24d), showing the superiority of pLink 2 both in sensitivity and precision. Finally, for cross-linked results obtained by pLink 2 in restricted database (Supplementary Fig. 24) and in *E. coli* whole proteome database (Fig. 3), the proportion of intersection set to union set is 91% and 87% respectively for PSMs and peptide pairs, showing the high consistency of pLink 2 when searching against the restricted database and the *E. coli* whole proteome database.

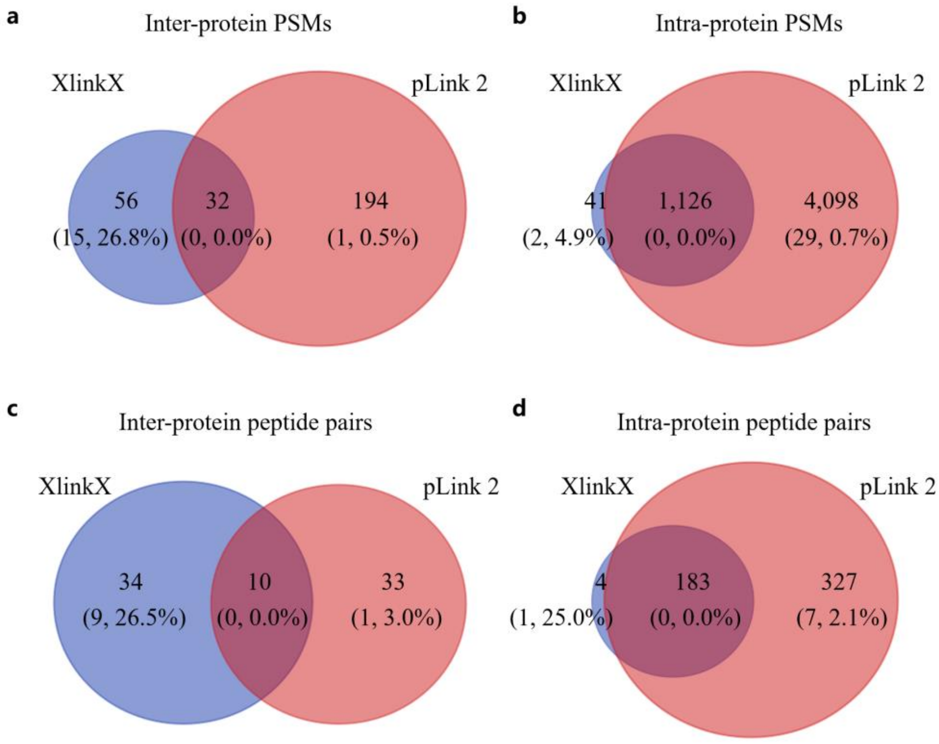

Supplementary Figure 24. Compare pLink 2 with XlinkX on the E.coli-Leiker-^{15}N dataset. At PSM level, the Venn diagram for **a)** inter-protein and **b)** intra-protein PSMs identified by XlinkX and pLink 2. Numbers in parentheses indicate the number and the percentage of NaN-ratio results that lie in the corresponding region. For example, 56 (15, 26.8%) means that XlinkX uniquely identified 56 inter-protein PSMs, of which 15 were NaN-ratios, accounting for 26.8% of 56. **c)** and **d)** are similar to a) and b) respectively, but at peptide pair level.

For the E.coli-SS-^{15}N dataset. Similarly, the database for XlinkX and pLink 2 searches contained only 1,181 proteins identified from RAW files of the sample using pFind. XlinkX identified a total of 6,19 cross-linked PSMs with 0% NaN-ratios, while pLink 2 identified sevenfold of that (4,448) with 1.0% NaN-ratios. Although XlinkX did not identify any NaN-ratio PSM, it identified many fewer cross-linked PSMs than pLink 2, and 96% of them were covered by pLink 2 (Supplementary Fig. 25), indicating the low sensitivity of XlinkX when analysing non-cleavable cross-linker data at a proteome scale of ~1,200 proteins.

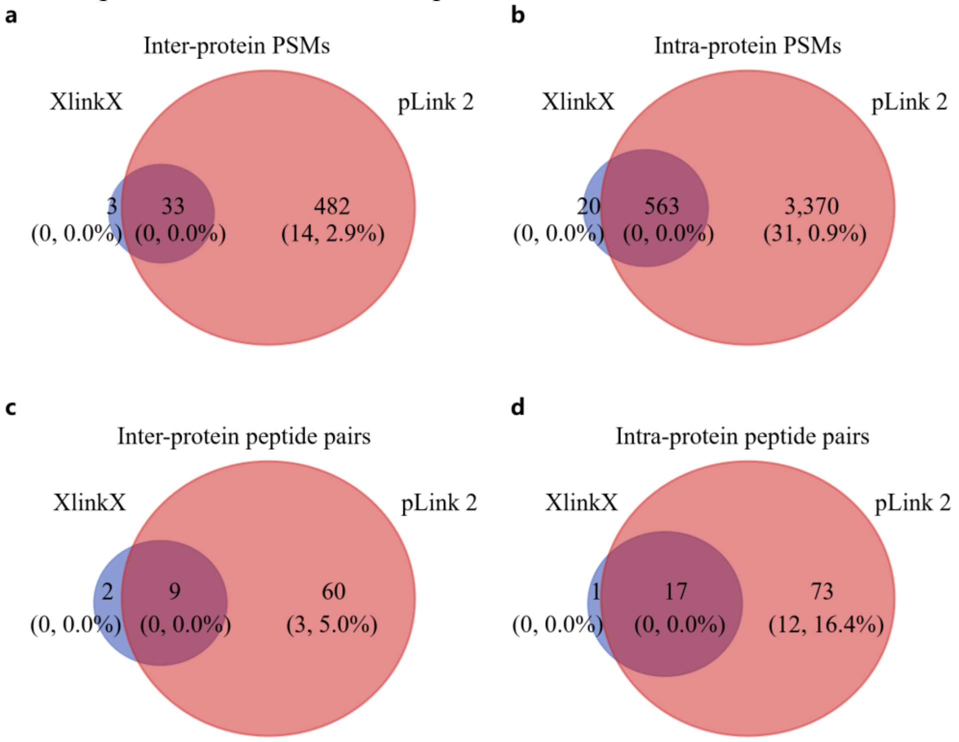

Supplementary Figure 25. Compare pLink 2 with XlinkX on the E.coli-SS-^{15}N dataset. At PSM level, the Venn diagram for **a)** inter-protein and **b)** intra-protein PSMs identified by XlinkX and pLink 2. **c)** and **d)** are similar to a) and b) respectively, but at peptide pair level.

Credibility evaluation using entrapment databases

The entrapment database method used in the main text took the intersection of cross-linked PSMs identified by Kojak, pLink 1, and pLink 2 as a new and fair standard dataset to search against entrapment databases of different sizes. However, the intersection spectra could not be a RAW file, thus could not be analysed by XlinkX. Nevertheless, XlinkX could search the original RAW files against entrapment databases, and the intersection PSMs identified by Kojak, pLink 1, and pLink 2 could serve as a ground truth.

Take the SCF(FBXL3)-BS3 dataset as an example. XlinkX searched three original RAW files against the original database plus increasing number of proteins from the *E. coli* database. As XlinkX only supports up to 1,500 proteins when analysing non-cleavable cross-linker data, the entrapment database for XlinkX search contained at most 1,000 proteins from the *E. coli* database. Furthermore, XlinkX threw an "OutOfMemoryError" exception when five variable modifications were set as

Supplementary Table 6, thus only Oxidation[M] was set as a variable modification. All other parameters were the same as those in Supplementary Table 6. Among the 850 cross-linked PSMs consistently identified by Kojak, pLink 1, and pLink 2 (Fig. 4 in the main text), 846 PSMs were not modified or modified only by Carbamidomethyl[C] or Oxidation[M]. Therefore, we took the 846 PSMs as a new and fair standard dataset (named intersection-846) to assess sensitivity and precision of XlinkX when searching against entrapment databases of different sizes. An identification was deemed correct if it is identical to an annotated PSM in the intersection-846; otherwise it was considered incorrect.

Supplementary Fig. 26 shows that although the sensitivities and precisions of pLink 2 and XlinkX kept stable along the entrapment database size increased, the sensitivity of XlinkX was less than 40%, much lower than that of pLink 2 (~99%).

For the Ca$_V$1.1-SS dataset, all parameters were the same as those in Supplementary Table 6, including two variable modifications. Therefore, we took the 836 PSMs consistently identified by Kojak, pLink 1, and pLink 2 as a new and fair standard dataset (named intersection-836, same as in Supplementary Fig. 12a) to assess sensitivity and precision of XlinkX when searching against entrapment databases of different sizes. Evaluation results were similar to those obtained for the SCF(FBXL3)-BS3 dataset (Supplementary Fig. 27). A possible reason for the stable sensitivity and precision might be that the size of entrapment database was too small, and if a whole proteome database was added as an entrapment database, more evident changes might be seen (Fig. 4 in the main text).

a

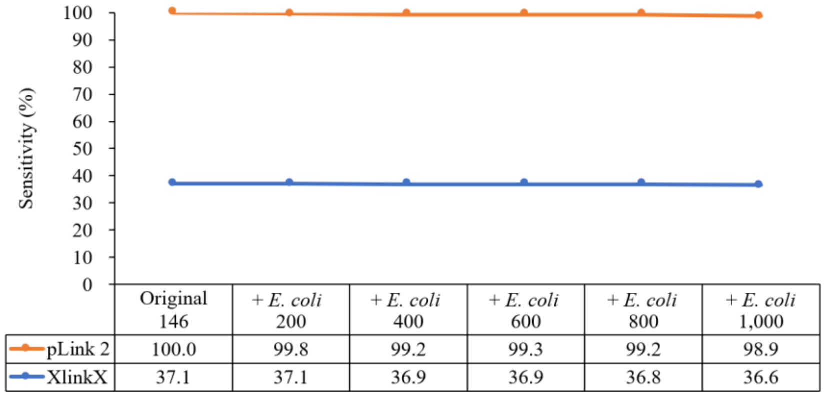

	Original 146	+ *E. coli* 200	+ *E. coli* 400	+ *E. coli* 600	+ *E. coli* 800	+ *E. coli* 1,000
pLink 2	100.0	99.8	99.2	99.3	99.2	98.9
XlinkX	37.1	37.1	36.9	36.9	36.8	36.6

b

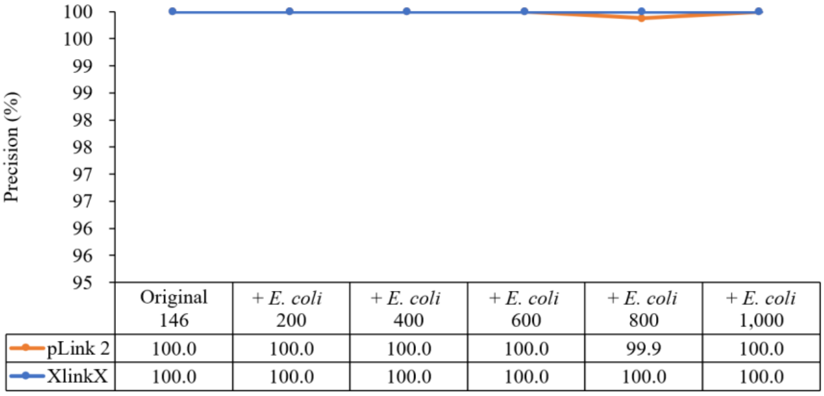

	Original 146	+ *E. coli* 200	+ *E. coli* 400	+ *E. coli* 600	+ *E. coli* 800	+ *E. coli* 1,000
pLink 2	100.0	100.0	100.0	100.0	99.9	100.0
XlinkX	100.0	100.0	100.0	100.0	100.0	100.0

Supplementary Figure 26. Performance evaluation on the SCF(FBXL3)-BS3 dataset. The **a)** sensitivity and **b)** precision of pLink 2 and XlinkX when searching three original RAW files against the original database plus increasing number of proteins from the *E. coli* database. The 846 PSMs consistently identified by Kojak, pLink 1, and pLink 2 were used as a new and fair standard dataset to assess sensitivity and precision.

a

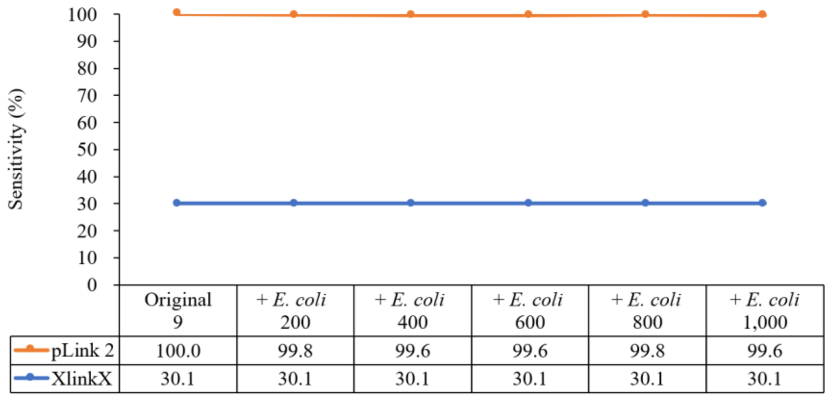

	Original 9	+ *E. coli* 200	+ *E. coli* 400	+ *E. coli* 600	+ *E. coli* 800	+ *E. coli* 1,000
pLink 2	100.0	99.8	99.6	99.6	99.8	99.6
XlinkX	30.1	30.1	30.1	30.1	30.1	30.1

b

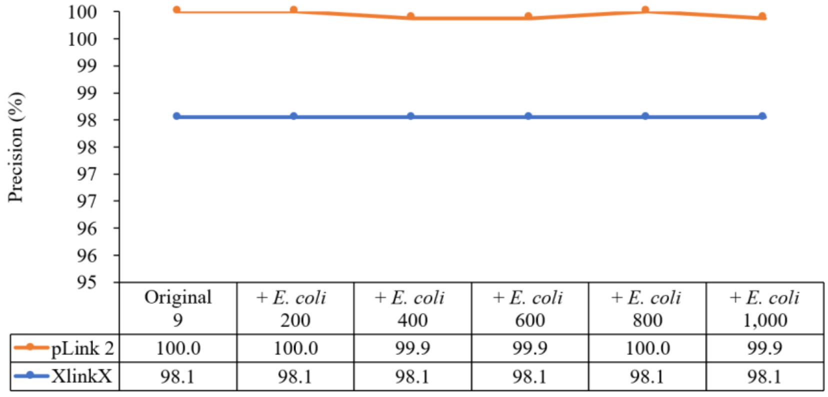

	Original 9	+ *E. coli* 200	+ *E. coli* 400	+ *E. coli* 600	+ *E. coli* 800	+ *E. coli* 1,000
pLink 2	100.0	100.0	99.9	99.9	100.0	99.9
XlinkX	98.1	98.1	98.1	98.1	98.1	98.1

Supplementary Figure 27. Performance evaluation on the Ca_V1.1-SS dataset. The **a)** sensitivity and **b)** precision of pLink 2 and XlinkX when searching two original RAW files against the original database plus increasing number of proteins from the *E. coli* database. The 836 PSMs consistently identified by Kojak, pLink 1, and pLink 2 were used as a new and fair standard dataset to assess sensitivity and precision.

Speed evaluation of XlinkX

XlinkX and pLink 2 were also compared in terms of computing time. Where possible, 8 threads were used for each comparison. The normalized computing times for XlinkX and pLink 2 (Windows 10, Intel Xeon E3-1241 CPU with 8 cores, 3.5 GHz, 16 GB RAM) are shown in Supplementary Table 9. On average, pLink 2 was 30 times faster than XlinkX when analysing data from non-cleavable cross-linkers. For benchmarking in the entrapment database method, the speedup of pLink 2 increased as the entrapment database size increased, showing the high efficiency of pLink 2, especially at a proteome scale.

Supplementary Table 9. The normalized computing times of XlinkX and pLink 2 on four datasets

Dataset	XlinkX	pLink 2	Real time[a]
E.coli-Leiker-^{15}N	22.7	1.0	67.4
E.coli-SS-^{15}N	10.4	1.0	65.2
SCF(FBXL3)-BS3 + Original	31.4	1.0	12.2
SCF(FBXL3)-BS3 + *E.coli* 200	34.6	1.0	19.2
SCF(FBXL3)-BS3 + *E.coli* 400	36.2	1.0	26.2
SCF(FBXL3)-BS3 + *E.coli* 600	36.9	1.0	32.2
SCF(FBXL3)-BS3 + *E.coli* 800	43.9	1.0	33.9
SCF(FBXL3)-BS3 + *E.coli* 1000	49.3	1.0	37.2
Cav1.1-SS + Original	40.0	1.0	2.9
Cav1.1-SS + *E.coli* 200	18.2	1.0	12.6
Cav1.1-SS + *E.coli* 400	20.4	1.0	16.8
Cav1.1-SS + *E.coli* 600	23.1	1.0	23.5
Cav1.1-SS + *E.coli* 800	25.1	1.0	28.5
Cav1.1-SS + *E.coli* 1000	27.1	1.0	35.5
Average	29.9	1.0	-

a The real search times for pLink 2 are shown in minutes.

Supplementary Note 5. Evaluate the performance of MassSpecStudio 2

MassSpecStudio 2 is another search engine for cross-linked peptide identification[25], and it employed a pre-scoring on linear peptides to reduce the search space of cross-linked peptide combinations. Its peptide library reduction strategy was similar to the open search strategy that had been widely used in pLink 1[4], Protein Prospector[6], and Kojak[7]. Similar to XlinkX, MassSpecStudio 2 could not analyse MGF files, and thus could not be evaluated using simulated datasets or synthetic datasets. Furthermore, MassSpecStudio 2 only reported identifications at peptide level, and thus could not be evaluated using entrapment databases like XlinkX in Supplementary Note 4. We finally evaluated the performance of MassSpecStudio 2 using ^{15}N metabolically labeled datasets. The search parameters for MassSpecStudio 2 were the same as in Supplementary Table 5 except that the FDR threshold was unknown and could not be set in MassSpecStudio 2. For each identified cross-linked peptide pair, MassSpecStudio 2 reported only one "Best MS2 Scan Number", we thus used that MS2 scan to calculate the ^{15}N quantitation ratio.

For the E.coli-Leiker-^{15}N dataset, as MassSpecStudio 2 was very slow, the database for MassSpecStudio 2 search contained only 1,284 proteins identified from the samples using pFind (same as in Supplementary Note 4). pLink 2 also searched against the same restricted database of 1,284 proteins, so that results could be fairly compared. MassSpecStudio 2 identified 242 inter-protein peptide pairs and 201 intra-protein peptide pairs (Supplementary Fig. 28), and this phenomenon was highly abnormal since intra-protein cross-links are more readily observed than inter-protein cross-links and most search engines identified more intra-proteins than inter-proteins (Fig. 3 in main text). Additionally, both inter-protein and intra-protein peptide pairs uniquely identified by MassSpecStudio 2 had much higher percentage of NaN-ratios compared with those uniquely identified by pLink 2. For the E.coli-SS-^{15}N dataset, similarly, the database for MassSpecStudio 2 and pLink 2 searches contained only 1,181 proteins identified from the samples using pFind. Evaluation results were similar to those obtained for the E.coli-Leiker-^{15}N dataset (Supplementary Fig. 29).

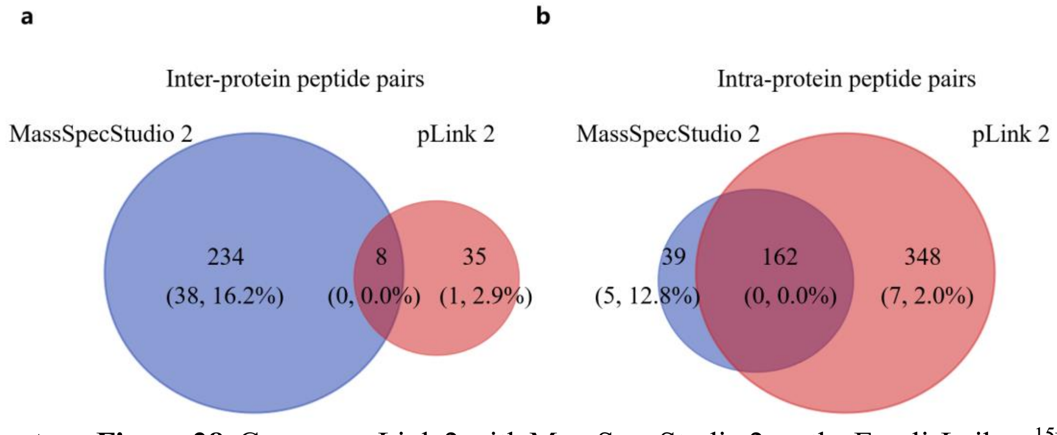

Supplementary Figure 28. Compare pLink 2 with MassSpecStudio 2 on the E.coli-Leiker-^{15}N dataset. At peptide pair level, the Venn diagram for **a)** inter-protein and **b)** intra-protein peptide pairs identified by MassSpecStudio 2 and pLink 2. Numbers in parentheses indicate the number and the percentage of NaN-ratio results that lie in the corresponding region. For example, 234 (38, 16.2%) means that MassSpecStudio 2 uniquely identified 234 inter-protein peptide pairs, of which 38 were NaN-ratios, accounting for 16.2% of 234.

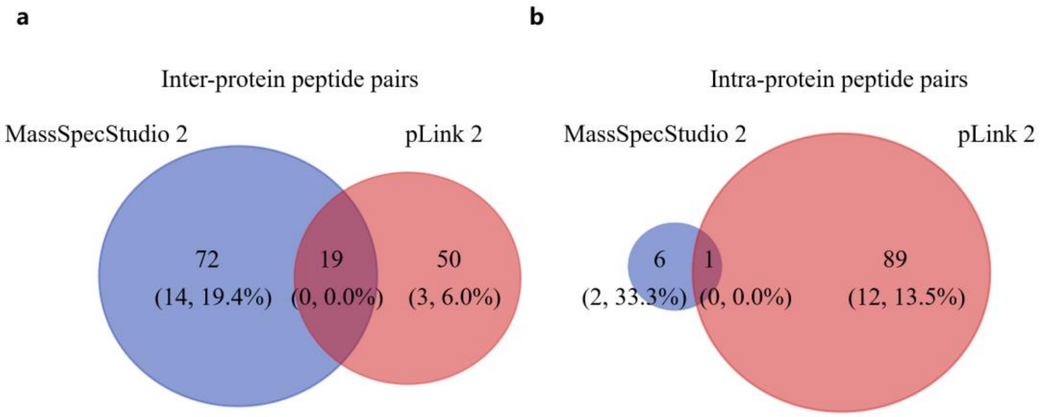

Supplementary Figure 29. Compare pLink 2 with MassSpecStudio 2 on the E.coli-SS-^{15}N dataset. At peptide pair level, the Venn diagram for **a)** inter-protein and **b)** intra-protein peptide pairs identified by MassSpecStudio 2 and pLink 2.

MassSpecStudio 2 and pLink 2 were also compared in terms of computing time. Where possible, 8 threads were used for each comparison. The computing times for MassSpecStudio 2 and pLink 2 (Windows Server, Intel Xeon E5-2670 CPU with 32 cores, 2.6 GHz, 128 GB RAM) are shown in Supplementary Fig. 30. With restricted databases, pLink 2 took only 1 hour to finish searching the E.coli-Leiker-^{15}N dataset or the E.coli-SS-^{15}N dataset, while MassSpecStudio 2 took more than 200 or more than 300 hours respectively, showing the high efficiency of pLink 2 at a proteome scale.

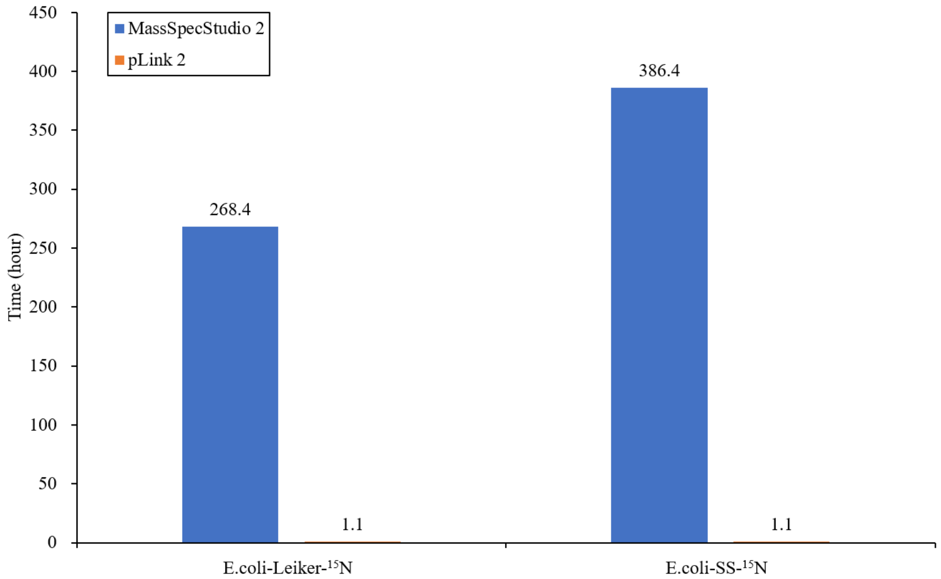

Supplementary Figure 30. The computing times for MassSpecStudio 2 and pLink 2 on the E.coli-Leiker-^{15}N dataset and the E.coli-SS-^{15}N dataset. Both MassSpecStudio 2 and pLink 2 searched against restricted databases containing only 1,284 proteins and 1,181 proteins identified by pFind for the E.coli-Leiker-^{15}N dataset and the E.coli-SS-^{15}N dataset respectively.

Supplementary Note 6. Evaluate the acceleration effect of fragment indexing

As described in the introduction of the main text, finding the top-*k* coarse-scored candidates is a new compute-intensive task in the open search strategy, as it has to examine all peptides whose masses are less than the precursor mass. Software performance profiling of pLink 1 showed that coarse-scoring took 71% of the total search time, thus representing a performance bottleneck (Supplementary Fig. 31). Although so many peptides were coarse-scored, a large proportion of them obtained a coarse-score of 0, *i.e.*, without even one matched fragment peak; they had to be coarse-scored just because their masses were less than the precursor mass.

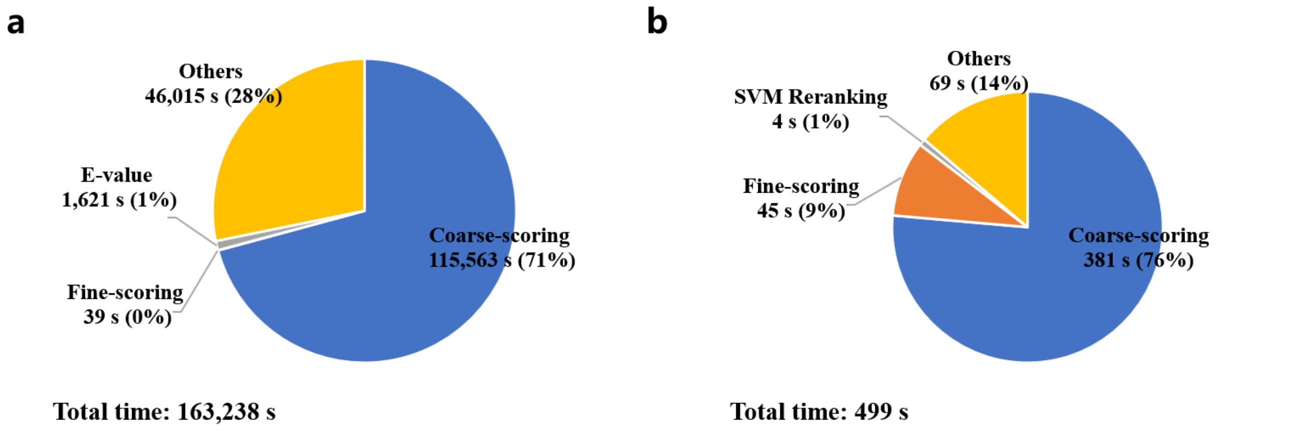

Supplementary Figure 31. Time cost analyses by searching the Synthetic-SS dataset using the *E. coli* entrapment database. **a)** For pLink 1, "Others" contained coarse-scoring for loop-linked, mono-linked, and regular peptide spectra, which was replaced by only fine-scoring in pLink 2. **b)** For pLink 2, "Others" contained fragment index constructing, peptide index constructing, and spectrum preprocessing. Both search engines run with a single thread.

Supplementary Table 10 shows that when pLink 1 searched the Synthetic-BS3 dataset against the *E. coli* entrapment database, there were an average of 143,044 peptide candidates for coarse-scoring per spectrum, which was about half of all the lysine-containing peptides in the database, highlighting that the number of coarse-scored peptides in the original open search strategy was very large. Among these candidates, 33.9% did not have a single matched peak, indicating a potential opportunity to use fragment indexing to filter these candidates out and to thereby accelerate the coarse-scoring stage. Beyond the large number of totally unmatched peptide candidates, it is also notable that the percentage of peptides with only one matched peak was also very high (47.4%), and these peptides cannot be credible identifications, a situation that further suggested an improvement space for speedup via MIC filters. Thus, over 80% of coarse-scored peptides evaluated with pLink 1 were not credible and can be removed using MIC filters to accelerate the open search strategy. Similar phenomena were found when using the other entrapment databases and on the Synthetic-SS dataset (Supplementary Table 11).

Supplementary Table 10. The scale of the coarse-scored peptides in the pLink 1 open search using different databases (the Synthetic-BS3 dataset)

Synthetic-BS3[a]	+ *E. coli*	+ Worm	+ Human
# lysine containing peptides	2.87×10^5	2.29×10^6	3.17×10^6
# coarse-scored peptides per spectrum	1.43×10^5	1.34×10^6	1.79×10^6
% coarse-scored peptides per spectrum[b]	49.8%	58.4%	56.5%
# peptides (MIC=0) per spectrum	4.84×10^4	4.45×10^5	6.04×10^5
% peptides (MIC=0) per spectrum[c]	33.9%	33.3%	33.7%
# peptides (MIC=1) per spectrum	6.78×10^4	6.37×10^5	8.54×10^5
% peptides (MIC=1) per spectrum[d]	47.4%	47.7%	47.7%
% peptides (MIC≤1) per spectrum[e]	81.3%	81.0%	81.4%

a Dataset: 2,077 spectra from the Synthetic-BS3 dataset. Database: an *E. coli*, worm, or human entrapment database was added to the 38 synthetic peptide sequences. Proteins were digested with the parameters shown in Supplementary Table 4
b The percentage of # coarse-scored peptides per spectrum in # lysine containing peptides
c The percentage of # peptides (MIC=0) per spectrum in # coarse-scored peptides per spectrum
d The percentage of # peptides (MIC=1) per spectrum in # coarse-scored peptides per spectrum
e The percentage in **c** plus the percentage in **d**

Supplementary Table 11. The scale of the coarse-scored peptides in the pLink 1 open search using different databases (the Synthetic-SS dataset)

Synthetic-SS[a]	+ *E. coli*	+ Worm	+ Human
# cysteine containing peptides	6.48×10^5	8.85×10^6	1.70×10^7
# coarse-scored peptides per spectrum	2.47×10^5	5.76×10^6	1.16×10^7
% coarse-scored peptides per spectrum[b]	38.1%	65.1%	68.6%
# peptides (MIC=0) per spectrum	1.10×10^5	2.73×10^6	5.46×10^6
% peptides (MIC=0) per spectrum[c]	44.5%	47.4%	46.9%
# peptides (MIC=1) per spectrum	1.00×10^5	2.38×10^6	4.82×10^6
% peptides (MIC=1) per spectrum[d]	40.7%	41.4%	41.4%
% peptides (MIC≤1) per spectrum[e]	85.2%	88.8%	88.3%

a Dataset: 5,000 spectra from the Synthetic-SS dataset. Database: an *E. coli*, worm, or human entrapment database was added to the 72 synthetic peptide sequences. Proteins were digested with parameters shown in Supplementary Table 4
b The percentage of # coarse-scored peptides per spectrum in # cysteine containing peptides
c The percentage of # peptides (MIC=0) per spectrum in # coarse-scored peptides per spectrum
d The percentage of # peptides (MIC=1) per spectrum in # coarse-scored peptides per spectrum
e The percentage in **c** plus the percentage in **d**

The same Synthetic-BS3 dataset was searched to evaluate the performance of the fragment indexing strategy. As shown in Supplementary Table 12, the fragment indexing used in pLink 2 remarkably enhanced the search speed by reducing the number of coarse-scored peptides. For example, when pLink 2 searched the Synthetic-BS3 dataset against the *E. coli* entrapment database, the average number of coarse-scored peptides per spectrum decreased from 143,044 in pLink 1 (Supplementary Table 10) to 679 in pLink 2 (Supplementary Table 12), which significantly accelerated the search. Actually, the number of initial candidates per spectrum (5.96×10^4 peptides with MIC ≥ 1) was already smaller than the number of coarse-scored peptides per spectrum in pLink 1 (1.43×10^5, Supplementary Table 10). Filtering out the peptide candidates with MIC = 1 (4.50×10^4 peptides, 75.6% of the total peptides with MIC ≥ 1) had almost no effect on sensitivity but significantly improved search efficiency. Further removing peptides whose mass was larger than the precursor mass and those whose MICs were fewer than the least MIC of the five candidates in the dynamic list (see Methods of the main text, Coarse-scoring on α-peptides) reduced the number of

final coarse-scored peptides to only 679 per spectrum, which was 1.1% of the total peptides with MIC ≥ 1 and 0.5% of the coarse-scored peptides in pLink 1. Using other entrapment databases and on the Synthetic-SS dataset, the same conclusion was reached (Supplementary Table 13).

Supplementary Table 12. Speed acceleration in pLink 2 by reducing the number of coarse-scored α-peptides without losing sensitivity (the Synthetic-BS3 dataset)

Synthetic-BS3[a]	+ *E. coli*	+ Worm	+ Human
# lysine containing peptides	2.87×10^{5}	2.29×10^{6}	3.17×10^{6}
# peptides (MIC≥1) per spectrum	5.96×10^{4}	6.72×10^{5}	1.16×10^{6}
% peptides (MIC≥1) per spectrum[b]	20.7%	29.4%	36.6%
# peptides (MIC=1) per spectrum	4.50×10^{4}	5.16×10^{5}	8.93×10^{5}
% peptides (MIC=1) per spectrum[c]	75.6%	76.8%	76.9%
# coarse-scored peptides per spectrum	6.79×10^{2}	1.88×10^{3}	2.23×10^{3}
% coarse-scored peptides per spectrum[d]	1.1%	0.3%	0.2%
Coarse-scoring: # pLink 2 / # pLink 1[e]	0.5%	0.1%	0.1%
Sensitivity (top-5)	99.9%	99.2%	98.6%

a The dataset, database, and search parameters were the same as in Supplementary Table 10
b The percentage of # peptides (MIC≥1) per spectrum in # lysine containing peptides
c The percentage of # peptides (MIC=1) per spectrum in # peptides (MIC≥1) per spectrum
d The percentage of # coarse-scored peptides per spectrum in # peptides (MIC≥1) per spectrum
e The ratio of # coarse-scored peptides per spectrum in pLink 2 to that in pLink 1 (Supplementary Table 10)

Supplementary Table 13. Speed acceleration in pLink 2 by reducing the number of coarse-scored α-peptides without losing sensitivity (the Synthetic-SS dataset)

Synthetic-SS[a]	+ *E. coli*	+ Worm	+ Human
# cysteine containing peptides	6.48×10^{5}	8.85×10^{6}	1.70×10^{7}
# peptides (MIC≥1) per spectrum	1.24×10^{5}	2.26×10^{6}	4.97×10^{6}
% peptides (MIC≥1) per spectrum[b]	19.2%	25.5%	29.3%
# peptides (MIC=1) per spectrum	9.68×10^{4}	1.79×10^{6}	3.95×10^{6}
% peptides (MIC=1) per spectrum[c]	77.8%	79.3%	79.5%
# coarse-scored peptides per spectrum	1.63×10^{3}	7.83×10^{3}	1.27×10^{4}
% coarse-scored peptides per spectrum[d]	1.3%	0.3%	0.3%
Coarse-scoring: # pLink 2 / # pLink 1[e]	0.7%	0.1%	0.1%
Sensitivity (top-5)	100.0%	99.7%	99.7%

a The dataset, database, and search parameters were the same as in Supplementary Table 11
b The percentage of # peptides (MIC≥1) per spectrum in # cysteine containing peptides
c The percentage of # peptides (MIC=1) per spectrum in # peptides (MIC≥1) per spectrum
d The percentage of # coarse-scored peptides per spectrum in # peptides (MIC≥1) per spectrum
e The ratio of # coarse-scored peptides per spectrum in pLink 2 to that in pLink 1 (Supplementary Table 11)

As shown in Supplementary Tables 12 and 13, although a large number of peptides were filtered out by the fragment indexing, the sensitivity for α-peptides in top-5 reached at least 98.6%. In the subsequent step of retrieving β-peptides, all peptide candidates whose masses matched to the open mass of α-peptide were retrieved and all combinations of α-β peptide pairs were fine-scored against the spectrum. As a result, there was little risk of losing the correct peptide pair candidate. Supplementary Figs. 2 and 4 show that the sensitivity of pLink 2 reached at least 97% even with a huge human entrapment database. It enlightens us that proper pruning did not affect sensitivity, and exhaustive search is not a necessity.

Software performance profiling of pLink 1 and pLink 2 (Supplementary Fig. 31) also show that the semi-supervised learning algorithm used in pLink 2 had greatly speeded up the PSM reranking procedure, as E-value calculation used in pLink 1 required additional 5,000 times fine-scoring of random sequence pairs for each PSM, which was time-consuming especially when the number of spectra was very large.

Supplementary Note 7. Application of pLink 2 at a proteome scale

To demonstrate the versatility and performance of pLink 2 at a proteome scale, pLink 2 was used to reanalyse four previously published datasets including *E. coli* and *C. elegans* whole-cell lysates cross-linked by Leiker, a BS3-like linker with enrichment function[15], and the *E. coli* and human disulfide proteomes[5]. In the original publications, PSM validations by TDA-FDR control were different for these two types of datasets, *i.e.*, global FDR control for intra-protein and inter-protein PSMs in combination was applied to two Leiker datasets, and separate FDR control was applied to two disulfide bond datasets. Parameters were adjusted to match those used in the published analysis so that results could be fairly compared. Results were shown in Supplementary Fig. 32.

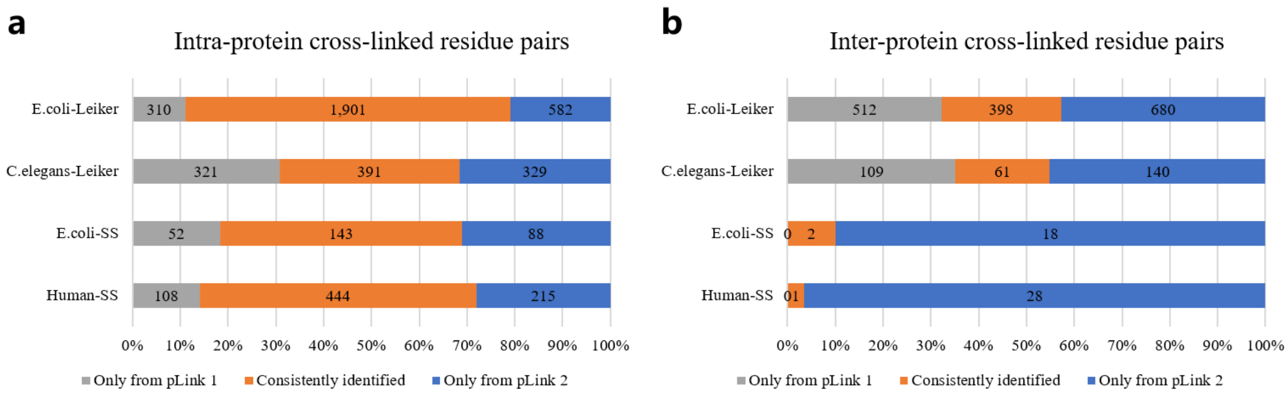

Supplementary Figure 32. Venn diagrams for the results of pLink 1 and pLink 2 on four previously published datasets. **a)** For the intra-protein cross-linked residue pairs. **b)** For the inter-protein cross-linked residue pairs. Results for pLink 1 were directly obtained from the original publications, where global FDR control for intra-protein and inter-protein PSMs was applied to two Leiker datasets, and separate FDR control was applied to two disulfide bond datasets. pLink 2 applied the same FDR control strategies as those of pLink 1 so that results could be fairly compared.

Reassuringly, the majority of residue pairs reported by two search engines were intra-protein cross-linked residue pairs, and for these results there were respectable overlaps in identifications (Supplementary Fig. 32a). However, there were poor overlaps for inter-protein cross-linked residue pairs (Supplementary Fig. 32b). For two Leiker datasets, both pLink 1 and pLink 2 used the global FDR control for intra-protein and inter-protein cross-linked identifications. As we have showed that the percentage of NaN-ratios of inter-protein PSMs reported by pLink 1 was much higher than that by pLink 2 under the global FDR control (Fig. 3, and Supplementary Fig. 9), the inter-protein results only from pLink 2 tended to be more credible.

Nevertheless, the separate FDR control is a more effective means to improve credibility of inter-protein results as demonstrated in Supplementary Note 2. Under the separate FDR control and with FDR set at 5% at the PSM level, pLink 2 identified 2,472 intra-protein cross-linked residue pairs and 389 inter-protein cross-linked residue pairs for the E.coli-Leiker dataset, and 723 intra-protein cross-linked residue pairs and 76 inter-protein cross-linked residue pairs for the C.elegans-Leiker dataset. For more details, please see Supplementary Data 1 and 2. For two disulfide bond datasets, both pLink 1 and pLink 2 used the separate FDR control, all inter-protein cross-linked residue pairs reported by pLink 1 were also reported by pLink 2, and more inter-protein cross-linked residue pairs were uniquely reported by pLink 2 (Supplementary Fig. 32b). For more details, please see Supplementary Data 3 and 4.

In summary, under the separate FDR control, pLink 2 identified 2,861 and 799 cross-linked residue pairs for E.coli-Leiker and C.elegans-Leiker datasets respectively, which were more credible than results of pLink 1 with the global FDR control; pLink 2 identified 251 and 688 cross-linked

residue pairs for E.coli-SS and Human-SS datasets respectively, which were 27% and 24% more than results of pLink 1 respectively.

Previously for pLink 1, large database search had been conducted on a computer cluster[4]. In the present study, all of the datasets were analysed on a personal computer. For each of the cell lysate dataset, the search time was several months for pLink 1, but 1~3 days for pLink 2. These results highlighted that pLink 2 is capable of analysing datasets cross-linked by a chemical cross-linker as well as native disulfide bonds, efficiently and effectively at a proteome scale.

Supplementary Note 8. The method used to generate simulated spectra

The Simulated-BS3 dataset consists of cross-linked, loop-linked, mono-linked, and regular MS2 spectra, 2,500 for each type, resulting in 10,000 MS2 spectra in total. As there are no mono-linked peptides in a disulfide bond sample, the Simulated-SS dataset only consists of 2,500, 2,500, and 5,000 for cross-linked, loop-linked, and regular MS2 spectra, respectively, resulting in 10,000 MS2 spectra in total.

Once the protein database, cross-linker, and modifications are specified (Supplementary Table 14), cross-linked MS2 spectra are generated in five steps as detailed below.

1. Each protein in the specified database is *in silico* enzymatically digested into peptides, and then modified regular peptides are generated according to user-defined modifications.
2. Two modified regular peptides, containing cross-linkable residues, are randomly selected as the α-peptide and the β-peptide.
3. Randomly assign a precursor charge state of +3, +4, +5, or +6 with probability 55%, 35%, 7%, or 3%, respectively. The charge distribution is obtained according to the synthetic datasets[4,5]. Hence, precursor m/z can be determined by combining the charge with α-peptide mass, β-peptide mass and linker mass.
    a. To better simulate the real situation, Gaussian error $e_1 \sim N(0, 3^2)$ ppm is added to the precursor m/z, and make sure that e_1 does not exceed 9.9 ppm.
4. Theoretical m/z of fragment ions b^{1+}, b^{2+}, y^{1+}, y^{2+} are calculated. The intensity of each ion type is set according to the average intensity of this ion type on the synthetic datasets (Average intensity in the Supplementary Fig. 33d). These theoretical ions with full m/z and intensity information make up the main fragment ion peaks. To better simulate the real situation, four types of noises are added to each fragment ion peak:
    a. A fragment ion peak is added with occurrence probability $\mathrm{Prob}_{\mathrm{occur}}$ in Supplementary Fig. 33d.
    b. Gaussian error $e_2 \sim N(0, 3^2)$ ppm is added to the m/z of the peak.
    c. Add one noise peak with probability 20%. The m/z of the noise peak equals to the m/z of the fragment ion peak adding Gaussian error $e_3 \sim N(0, 6.5^2)$ ppm, the intensity of the noise peak equals to 10% of the intensity of the fragment ion peak.
    d. The first and second isotopic peaks of the fragment ion peak are added to the spectrum to help search engines determine the charge state of the fragment ion peak. The intensities of isotopic peaks are calculated using the EMASS algorithm[26].
5. Finally, merge adjacent peaks with approximately the same m/z ($\triangle$m/z < 1E-5 Th).

In Step 4a, we calculate the occurrence probabilities ($\mathrm{Prob}_{\mathrm{occur}}$) of ion types b^{1+}, b^{2+}, y^{1+}, and y^{2+} for the Synthetic-BS3 dataset (Supplementary Fig. 33a) and the Synthetic-SS dataset (Supplementary Fig. 33b). The $\mathrm{Prob}_{\mathrm{occur}}$ is the number of matched peaks of an ion type divided by the total number of theoretical peaks of that ion type, which means the occurrence probability of that ion type. On average, y^{1+} has the highest occurrence probability (0.60), while the occurrence probabilities of other three ion types lie between 0.25 and 0.33 (Supplementary Fig. 33c). In order to generate higher-quality simulated spectra, we increase the occurrence probability of y^{1+} to 0.8, and increase the occurrence probabilities of other three ion types to 0.5 (Supplementary Fig. 33d), making the simulated spectra contain more fragment ion peaks than the spectra of synthetic peptides.

Supplementary Fig. 34 shows two examples of simulated BS3 and SS cross-linked spectra. Loop-linked, mono-linked, and regular spectra are generated in the similar way except that only one modified peptide needed to be randomly selected from all modified peptides in step 2.

As both α- and β- peptides are theoretically fragmented better than synthetic peptides, not containing any complicated ions such as internal ions, the simulated spectra are rather simpler compared with the real-world spectra. In our study, we firstly use simulated datasets to evaluate ten established cross-linked peptide search engines, and only those passing this qualification test will proceed to participate in the following comparisons.

The source code of the simulation method used to generate simulated spectra, termed as pSimXL, is publicly available at GitHub: https://github.com/pFindStudio/pLink2/tree/master/pSimXL. Anyone can review and download the source code of pSimXL under the open source GNU General Public License v3.0. More importantly, any search engine for identification of cross-linked peptides can use pSimXL to debug and improve performance. We believe that pSimXL will be highly beneficial to the CXMS community.

Supplementary Table 14. The parameters used for generating and searching two simulated datasets

Items	Settings
Database	First 100 proteins in *E. coli* database
Spectra	10,000 spectra (2,500 cross-linked spectra and 7,500 *non*-cross-linked spectra)
Cross-linker	BS3 for Simulated-BS3 and SS for Simulated-SS
Enzyme	Trypsin
Max Missed Cleavage Sites	2
Peptide Mass Range	[600, 6,000] Da
Peptide Length Range	[6,60]
Precursor Tolerance	± 10 ppm
Fragment Tolerance	± 20 ppm
Fixed Modifications	Carbamidomethylation (C) for Simulated-BS3
Variable Modifications	Oxidation (M) for Simulated-BS3 and Nethylmaleimide(C) for Simulated-SS
Max Modified Sites	3

a Synthetic-BS3

Ion type	$Prob_{occur}$	Average intensity
b^{1+}	0.19	0.12
b^{2+}	0.23	0.08
y^{1+}	0.71	0.22
y^{2+}	0.37	0.14

b Synthetic-SS

Ion type	$Prob_{occur}$	Average intensity
b^{1+}	0.30	0.10
b^{2+}	0.24	0.06
y^{1+}	0.48	0.20
y^{2+}	0.29	0.09

c Average of Synthetic-BS3 and Synthetic-SS

Ion type	$Prob_{occur}$	Average intensity
b^{1+}	0.25	0.11
b^{2+}	0.24	0.07
y^{1+}	0.60	0.21
y^{2+}	0.33	0.12

d Simulated-BS3 and Simulated-SS

Ion type	$Prob_{occur}$	Average intensity
b^{1+}	0.50	0.11
b^{2+}	0.50	0.07
y^{1+}	0.80	0.21
y^{2+}	0.50	0.12

Supplementary Figure 33. The fragmentation characteristics of synthetic datasets and simulated datasets. **a)** The occurrence probabilities and average intensities of ion types b^{1+}, b^{2+}, y^{1+}, and y^{2+} for the Synthetic-BS3 dataset. The occurrence probability is the number of matched peaks of an ion type divided by the total number of theoretical peaks of that ion type. The average intensity is the average of normalized intensity of matched peaks belonging to the same ion type (normalized to the base peak intensity). **b)** Similar to a), but for the Synthetic-SS dataset. **c)** The average of a) and b). **d)** The occurrence probabilities and average intensities used to generate simulated spectra. Compared with synthetic datasets, the occurrence probability of y^{1+} is increased to 0.8 and the occurrence probabilities of other three ion types are increased to 0.5, making the simulated spectra contain more fragment ion peaks than the spectra of synthetic peptides.

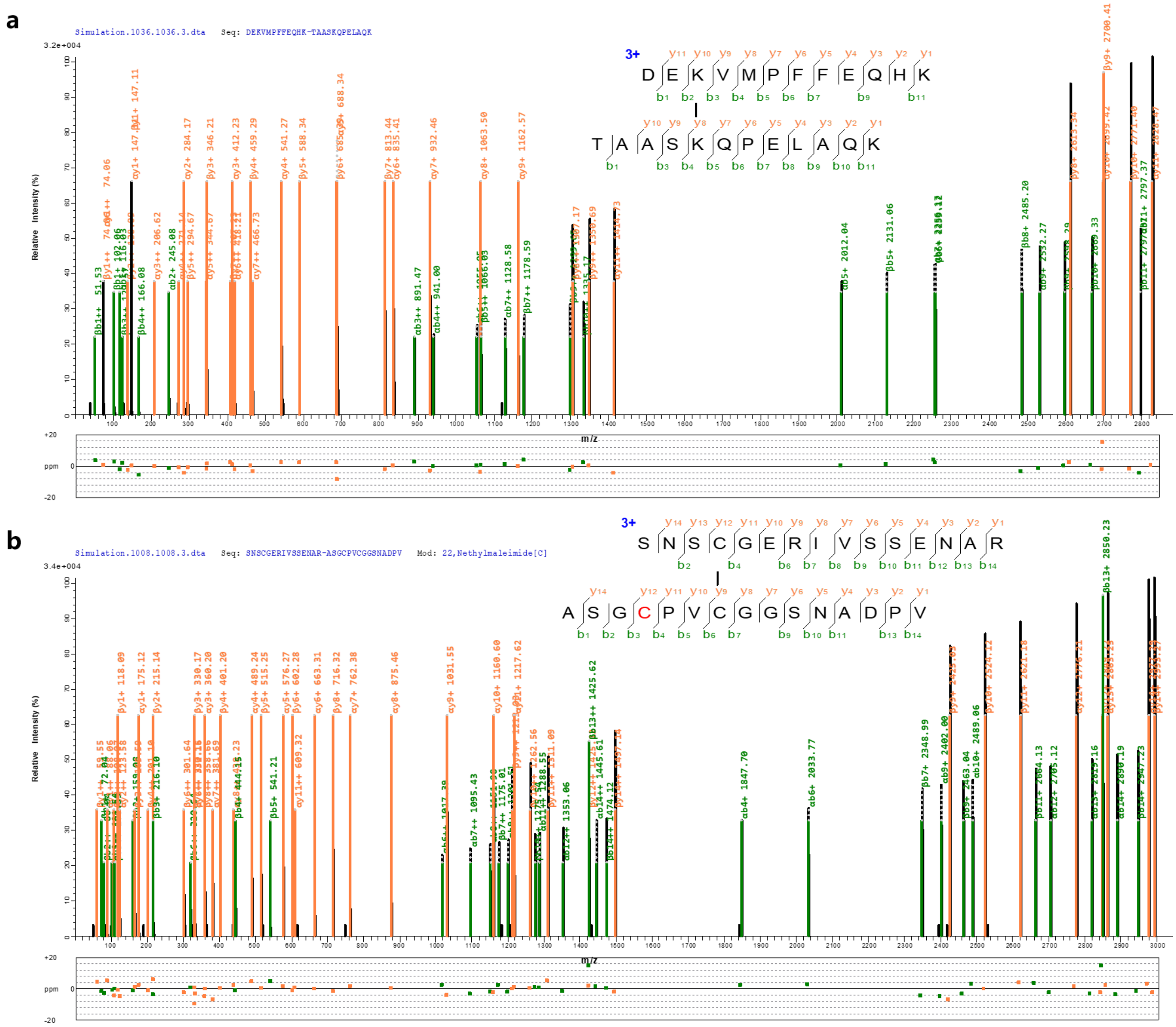

Supplementary Figure 34. Two examples of simulated spectra cross-linked by **a)** BS3 and **b)** disulfide bond respectively and annotated by pLabel.

SUPPLEMENTARY REFERENCES

1 Rinner, O. *et al.* Identification of cross-linked peptides from large sequence databases (vol 5, pg 315, 2008). *Nat Methods* **5**, 748-748 (2008).

2 Walzthoeni, T. *et al.* False discovery rate estimation for cross-linked peptides identified by mass spectrometry. *Nat Methods* **9**, 901-903, doi:10.1038/nmeth.2103 (2012).

3 Gotze, M. *et al.* StavroX-A Software for Analyzing Crosslinked Products in Protein Interaction Studies. *J Am Soc Mass Spectr* **23**, 76-87 (2012).

4 Yang, B. *et al.* Identification of cross-linked peptides from complex samples. *Nat Methods* **9**, 904-+ (2012).

5 Lu, S. *et al.* Mapping native disulfide bonds at a proteome scale. *Nat Methods* **12**, 329-U373 (2015).

6 Trnka, M. J., Baker, P. R., Robinson, P. J. J., Burlingame, A. L. & Chalkley, R. J. Matching Cross-linked Peptide Spectra: Only as Good as the Worse Identification. *Mol Cell Proteomics* **13**, 420-434 (2014).

7 Hoopmann, M. R. *et al.* Kojak: Efficient Analysis of Chemically Cross-Linked Protein Complexes. *J Proteome Res* **14**, 2190-2198 (2015).

8 Giese, S. H., Fischer, L. & Rappsilber, J. A Study into the Collision-induced Dissociation (CID) Behavior of Cross-Linked Peptides. *Mol Cell Proteomics* **15**, 1094-1104, doi:10.1074/mcp.M115.049296 (2016).

9 Mendes, M. L. *et al.* An integrated workflow for crosslinking mass spectrometry. *bioRxiv* (2019).

10 Yilmaz, S. *et al.* Xilmass: A New Approach toward the Identification of Cross-Linked Peptides. *Anal Chem* **88**, 9949-9957 (2016).

11 Lu, L. *et al.* Identification of MS-Cleavable and Noncleavable Chemically Cross-Linked Peptides with MetaMorpheus. *J Proteome Res* **17**, 2370-2376, doi:10.1021/acs.jproteome.8b00141 (2018).

12 Dai, J., Jiang, W., Yu, F., Yu, W. & Stegle, O. Xolik: finding cross-linked peptides with maximum paired scores in linear time. *Bioinformatics*, doi:10.1093/bioinformatics/bty526 (2018).

13 Meng, J. M. *et al.* in *Proceedings of the 65th ASMS Conference on Mass Spectrometry and Allied Topics.*

14 Wu, J. *et al.* Structure of the voltage-gated calcium channel Ca(v)1.1 at 3.6 A resolution. *Nature* **537**, 191-196, doi:10.1038/nature19321 (2016).

15 Tan, D. *et al.* Trifunctional cross-linker for mapping protein-protein interaction networks and comparing protein conformational states. *Elife* **5** (2016).

16 Fu, Y. & Qian, X. Transferred subgroup false discovery rate for rare post-translational modifications detected by mass spectrometry. *Mol Cell Proteomics* **13**, 1359-1368, doi:10.1074/mcp.O113.030189 (2014).

17 Liu, C. *et al.* pQuant Improves Quantitation by Keeping out Interfering Signals and Evaluating the Accuracy of Calculated Ratios. *Anal Chem* **86**, 5286-5294 (2014).

18 Zhou, W. J. *et al.* pValid: Validation Beyond the Target-Decoy Approach for Peptide Identification in Proteomics. *Under reivew.*

19 Alves, G., Wu, W. W., Wang, G., Shen, R. F. & Yu, Y. K. Enhancing peptide identification confidence by combining search methods. *J Proteome Res* **7**, 3102-3113, doi:10.1021/pr700798h (2008).

20 Dagda, R. K., Sultana, T. & Lyons-Weiler, J. Evaluation of the Consensus of Four Peptide Identification Algorithms for Tandem Mass Spectrometry Based Proteomics. *J Proteomics Bioinform* **3**, 39-47, doi:10.4172/jpb.1000119 (2010).

21 Shteynberg, D. *et al.* iProphet: Multi-level Integrative Analysis of Shotgun Proteomic Data Improves Peptide and Protein Identification Rates and Error Estimates. *Mol Cell Proteomics* **10** (2011).

22 Liu, F., Lossl, P., Scheltema, R., Viner, R. & Heck, A. J. R. Optimized fragmentation schemes and data analysis strategies for proteome-wide cross-link identification. *Nat Commun* **8** (2017).

23 Liu, F., Rijkers, D. T. S., Post, H. & Heck, A. J. R. Proteome-wide profiling of protein assemblies by cross-linking mass spectrometry. *Nat Methods* **12**, 1179-+ (2015).

24 Chi, H. *et al.* Comprehensive identification of peptides in tandem mass spectra using an efficient open search engine. *Nat Biotechnol*, doi:10.1038/nbt.4236 (2018).

25 Sarpe, V. *et al.* High Sensitivity Crosslink Detection Coupled With Integrative Structure Modeling in the Mass Spec Studio. *Mol Cell Proteomics* **15**, 3071-3080, doi:10.1074/mcp.O116.058685 (2016).

26 Rockwood, A. L. & Haimi, P. Efficient calculation of accurate masses of isotopic peaks. *J Am Soc Mass Spectrom* **17**, 415-419, doi:10.1016/j.jasms.2005.12.001 (2006).
