# Supplementary material for: A high-speed search engine pLink 2 with systematic evaluation for proteome-scale identification of cross-linked peptides
Source: Nat Commun. 2019 Jul 30;10:3404. doi: 10.1038/s41467-019-11337-z (PMC6667459; doi:10.1038/s41467-019-11337-z)
Supplement: Supplementary file 3 — Reporting summary [file 41467_2019_11337_MOESM3_ESM.pdf]

nature research

Corresponding author(s): Meng-Qiu Dong, Si-Min He

Last updated by author(s): Jun 10, 2019

Reporting Summary

Nature Research wishes to improve the reproducibility of the work that we publish. This form provides structure for consistency and transparency in reporting. For further information on Nature Research policies, see Authors & Referees and the Editorial Policy Checklist.

Statistics

For all statistical analyses, confirm that the following items are present in the figure legend, table legend, main text, or Methods section.

n/a	Confirmed	
☐	☒	The exact sample size (*n*) for each experimental group/condition, given as a discrete number and unit of measurement
☐	☒	A statement on whether measurements were taken from distinct samples or whether the same sample was measured repeatedly
☐	☒	The statistical test(s) used AND whether they are one- or two-sided<br>*Only common tests should be described solely by name; describe more complex techniques in the Methods section.*
☐	☒	A description of all covariates tested
☐	☒	A description of any assumptions or corrections, such as tests of normality and adjustment for multiple comparisons
☐	☒	A full description of the statistical parameters including central tendency (e.g. means) or other basic estimates (e.g. regression coefficient) AND variation (e.g. standard deviation) or associated estimates of uncertainty (e.g. confidence intervals)
☐	☒	For null hypothesis testing, the test statistic (e.g. *F*, *t*, *r*) with confidence intervals, effect sizes, degrees of freedom and *P* value noted<br>*Give P values as exact values whenever suitable.*
☐	☒	For Bayesian analysis, information on the choice of priors and Markov chain Monte Carlo settings
☐	☒	For hierarchical and complex designs, identification of the appropriate level for tests and full reporting of outcomes
☐	☒	Estimates of effect sizes (e.g. Cohen's *d*, Pearson's *r*), indicating how they were calculated

Our web collection on statistics for biologists contains articles on many of the points above.

Software and code

Policy information about availability of computer code

Data collection

1. A custom algorithm (https://github.com/pFindStudio/pLink2/tree/master/pSimXL) was used to generate two simulated datasets (Simulated-BS3 and Simulated-SS).

2. The EASY-nLC 1000 ultra-HPLC system (Thermo Fisher Scientific) coupled online to an Q Exactive™ HF mass spectrometer (Thermo Fisher Scientific) were used to collect two 15N metabolically labeled datasets (E.coli-Leiker-15N and E.coli-SS-15N). (Tune 2.8 SP1 build 2806, Xcalibur 4.0.27.19)

3. Other datasets were published previsouly, the detailed information is shown in Supplementary Table 2.

4. pXtract v2.0 (http://pfind.ict.ac.cn/software/pXtract/index.html) was used for converting RAW files to MS1 and MS2 files.

Data analysis

1. Twelve cross-linked peptide search engines were used in this study.
1) xQuest 2.1.1
2) StavroX 3.6.0.1
3) pLink 1.23
4) Protein Prospector v5.21.2
5) Kojak 1.5.5
6) Xi 1.6.731
7) Xilmass 1.0
8) MetaMorpheusXL 0.0.285
9) Xolik 0.3
10) pLink 2.2
11) XlinkX 2.3
12) MassSpecStudio 2

2. A semi-supervised learning algorithm was used to iteratively separate target PSMs from decoy ones based on the linear classification software package LIBLINEAR.

3. pQuant v2.0 (http://pfind.ict.ac.cn/software/pQuant/index.html) was used to check the quantitation ratio of each PSM identified.

For manuscripts utilizing custom algorithms or software that are central to the research but not yet described in published literature, software must be made available to editors/reviewers. We strongly encourage code deposition in a community repository (e.g. GitHub). See the Nature Research guidelines for submitting code & software for further information.

Data

Policy information about availability of data

All manuscripts must include a data availability statement. This statement should provide the following information, where applicable:

- Accession codes, unique identifiers, or web links for publicly available datasets
- A list of figures that have associated raw data
- A description of any restrictions on data availability

1. The simulated datasets are published along with the source code of the simulation method at GitHub: https://github.com/pFindStudio/pLink2/tree/master/pSimXL.

2. The RAW data is uploaded on PRIDE with following information.
Project accession: PXD012109
Web link: https://www.ebi.ac.uk/pride/archive/projects/PXD012109
Username: reviewer21162@ebi.ac.uk
Password: puL7ioYP

3. The project contains following files.
15 RAW files
1 FASTA file
2 Parameter files
2 identification result files

4. The data is currently private, and can only be accessed with the username and password provided above, it will be public when the manuscript is accepted.

Field-specific reporting

Please select the one below that is the best fit for your research. If you are not sure, read the appropriate sections before making your selection.

☒ Life sciences ☐ Behavioural & social sciences ☐ Ecological, evolutionary & environmental sciences

For a reference copy of the document with all sections, see nature.com/documents/nr-reporting-summary-flat.pdf

Life sciences study design

All studies must disclose on these points even when the disclosure is negative.

Sample size	Sample sizes were chosen based on preliminary experiments so as to provide sufficient power for statistical comparison (where appropriate).
Data exclusions	No data were excluded from the analyses.
Replication	The experimental findings in this manuscript are reliably reproduced. The sensitivity, precision and speed of pLink 2 are measured using twelve different datasets.
Randomization	This is not relevant to our study. For pLink 2 and other search engines, ALL MS/MS data extracted from RAW files are searched against protein databases.
Blinding	It is not relevant to our study because this study is mainly focused on the methods of MS/MS data analysis.

Reporting for specific materials, systems and methods

We require information from authors about some types of materials, experimental systems and methods used in many studies. Here, indicate whether each material, system or method listed is relevant to your study. If you are not sure if a list item applies to your research, read the appropriate section before selecting a response.

Materials & experimental systems

n/a	Involved in the study
☒	☐ Antibodies
☒	☐ Eukaryotic cell lines
☒	☐ Palaeontology
☒	☐ Animals and other organisms
☒	☐ Human research participants
☒	☐ Clinical data

Methods

n/a	Involved in the study
☒	☐ ChIP-seq
☒	☐ Flow cytometry
☒	☐ MRI-based neuroimaging
