# Supplementary material for: A high-speed search engine pLink 2 with systematic evaluation for proteome-scale identification of cross-linked peptides
Source: Nat Commun. 2019 Jul 30;10:3404. doi: 10.1038/s41467-019-11337-z (PMC6667459; doi:10.1038/s41467-019-11337-z)
Supplement: Supplementary file 4 — Description of Additional Supplementary Files [file 41467_2019_11337_MOESM4_ESM.docx]

**Description of Additional Supplementary Files**

File Name: Supplementary Data 1

Description: Reanalysis of the E.coli-Leiker dataset

File Name: Supplementary Data 2

Description: Reanalysis of the C.elegans-Leiker dataset

File Name: Supplementary Data 3

Description: Reanalysis of the E.coli-SS dataset

File Name: Supplementary Data 4

Description: Reanalysis of the Human-SS dataset
